# Navigating the potential of algal peptides: health effects, market applications, and scientific challenges

**DOI:** 10.1080/07853890.2026.2637282

**Published:** 2026-03-07

**Authors:** Muniba Khaliq, Zahra Noor, Mnahil Moazzam, Fatima Saeed, Khadija- Tul-Zohra, Eisha Tariq, Hafiz Muhammad Shahbaz, Kashaf Khaliq

**Affiliations:** a Department of Food and Nutritional Sciences, University of Reading, Berkshire, United Kingdom; b Department of Food Science and Human Nutrition, University of Veterinary and Animal Sciences, Lahore, Pakistan; c Nutrition Department, Allied Health Sciences, Akhtar Saeed Medical and Dental College, Lahore, Pakistan; d Department of Nutrition and Health, College of Medicine and Health Sciences, United Arab Emirates University, Al Ain, United Arab Emirates; e Institute of Pharmaceutical Sciences, University of Veterinary and Animal Sciences, Lahore, Pakistan

**Keywords:** Algal bioactive peptides, microalgae, functional foods, nutraceuticals, bioavailability, nanoencapsulation, sustainable nutrition

## Abstract

**Introduction:**

Algae-derived bioactive peptides are gaining recognition as functional ingredients offering health benefits and sustainability advantages over conventional proteins. This review aimed to evaluate the current evidence on algal peptides, focusing on their nutritional content, mechanistic actions, health effects, potential for sustainability, and translational challenges.

**Materials and Methods:**

A comprehensive literature search was conducted across PubMed, Scopus, ScienceDirect, Springer, Elsevier, and Google Scholar. Peer-reviewed studies reporting bioactive peptides derived from microalgae, macroalgae, or Cyanophyceae were included. *In vitro*, animal, and human intervention studies evaluating molecular mechanisms, metabolic outcomes, or clinical relevance were considered.

**Results:**

Available evidence shows that algal peptides exert multifunctional bioactivities, including inhibition of angiotensin-converting enzyme and renin, antioxidant and anti-inflammatory effects, modulation of glucose metabolism *via* α-amylase, α-glucosidase, and DPP-IV inhibition, and regulation of lipid metabolism and adipogenesis. Frequently studied sources included *Limnospira*, *Chlorella*, *Auxenochlorella*, *Nannochloropsis*, *Undaria*, *Palmaria*, *Ulva*, and *Neopyropia*. Limited human trials suggest modest but clinically relevant improvements in blood pressure, glycemic control, lipid profiles, and body-weight-related outcomes, primarily using whole algal biomass or extracts. Life-cycle assessments highlight favorable land-use efficiency and carbon sequestration potential, although economic feasibility is constrained by energy-intensive downstream processing.

**Conclusion:**

Algal-derived peptides demonstrate promising health-promoting effects and align with sustainable nutrition goals. However, their clinical translation is limited by variability in peptide characterization, uncertain bioavailability, and lack of robust human trials. Standardized production methods, improved delivery strategies, comprehensive safety assessments, and well-designed clinical studies are essential to support their application in functional foods and nutraceuticals.

## 1. Introduction

Algae are among the oldest organisms on earth, first appearing approximately 3.5 billion years ago [1]. Algae are regarded as “superfoods” due to their exceptional and diverse nutritional profile. This heterogeneous group comprises both unicellular microalgae and multicellular macroalgae, which exhibit distinct biological and ecological characteristics. They are recognized for their rapid growth, effective utilization of light energy, ability to fix atmospheric CO_2_, and capacity to produce greater biomass per hectare than vascular plants [2]. Microalgae, in particular, represent an exceptionally diverse group, with species estimates ranging from 200,000 to 800,000 [3]. They are among the most efficient photosynthetic organisms, with reported photosynthetic efficiencies of approximately 10–20%, exceeding those of most terrestrial plants under optimized conditions [4]. These attributes have positioned microalgae as promising candidates for sustainable biomass and protein production. However, productivity and efficiency vary widely among species and cultivation systems, and large-scale implementation remains influenced by technological, economic, and environmental constraints.

From a sustainability perspective, microalgae offer several advantages over land-based food systems [5]. Photosynthetic microalgae are found in every habitat and have wide ecological adaptations. Furthermore, microalgae cultivation mounts less stress on arable land and freshwater resources, as they can be grown on non-arable land with a minimum quantity of freshwater or even cultivated in wastewater or seawater [6]. Over recent decades, microalgae biomass has been widely used as an additive in aquatic and animal feed, highlighting its growing significance and practical applications [7].

In terms of land-use efficiency, algal proteins require substantially less land than both animal- and plant-derived protein sources. For instance, producing 1 kg of algal protein requires only about 2.5 m^2^ of land, whereas pork, chicken, and beef require approximately 47–64 m^2^, 42–52 m^2^, and 144–258 m^2^ of land, respectively [8,9]. Algae also demonstrate superior land efficiency over several plant-based sources, with nuts requiring 7.9 m^2^, pulses 7.3 m^2^, grains 4.6 m^2^, and peas 3.4 m^2^ of land per kg of protein [10].

Beyond protein quantity, microalgae are increasingly studied for their bioactive components, including peptides with potential health-promoting properties. As such, microalgae align closely with several Sustainable Development Goals (SDGs) [11], particularly those related to food security, health promotion, responsible consumption, and environmental sustainability [12]. Accordingly, this review consolidates current evidence on algal bioactive peptides, with a critical emphasis on mechanistic insights, preclinical outcomes, and emerging clinical data. In parallel, it addresses sustainability considerations, technological innovations, and regulatory challenges that collectively shape the future development and application of algal peptides in food and health sectors.

### 1.1. The evolution of microalgae

Microalgae emerged billions of years ago and have been a fundamental food source for fish and other aquatic animals [13]. For centuries, microalgae have served as a crucial food source for humans. Populations in regions such as Asia and North America have traditionally relied on microalgae biomass, both as a source of nourishment and as a potent nutritional supplement [1]. In 1300 AD, the Aztecs in Mexico harvested cyanobacterium *Limnospira platensis* (formerly *Arthrospira platensis*, commercially known as *Spirulina*) from Lake Texcoco as a crucial famine food [1]. The first significant mass cultures were created by the Japanese post-World War II, highlighting microalgae’s potential as a vital alternative protein source during times of food scarcity [1]. Moreover, the filamentous cyanobacterium *Nostoc flagelliforme* has been a staple in the Chinese diet for over 2,000 years [1,2]. The modern era of microalgal biotechnology emerged in the early 1940s and gained significant advancement in 1952 with the Algal Mass-Culture Symposium at Stanford University, which introduced various techniques for mass-controlled cultivation, including open ponds and photobioreactors [2]. Commercial microalgae production commenced in Japan in the 1960s with the cultivation of *Chlorella* (Chlorophyta), followed by *Spirulina* (Cyanophyceae) farming in Mexico, USA, and India in the 1970s. In India, a joint initiative between India and Germany was launched in 1973 at the Central Food Technological Research Institute (CFTRI) to promote the mass production of algae for protein. Initially, the research centered on *Tetradesmus obliquus* (formerly *Scenedesmus obliquus*) (Chlorophyta), but the focus transitioned to *Spirulina* (Cyanophyceae) due to the difficulties encountered in harvesting the former species, making *Spirulina/Limnospira* a more practical choice [14]. In the 1970s, single-cell protein (SCP) derived from microalgal biomass emerged as a significant product in the market, primarily for use in food and nutritional supplements [15]. In 1974, the United Nations World Food Conference highlighted *Spirulina* as a future food due to its rich iron and protein content and its suitability for children [2]. By 2003, the FAO reported significant *Spirulina* production in China, underscoring its global rise as a nutraceutical. The FAO further endorsed *Spirulina* in 2008 as a promising solution for combating malnutrition [16]. Microalgae are currently utilized across various industries, including human nutrition, animal feed, fertilizers, and as ingredients in cosmetic and pharmaceutical products [17,18]. Additionally, genetic engineering can be used to modify microalgal species, enhancing their diversity and competitiveness [19].

### 1.2. Nutritional profile and quality assessment of algal peptides

Algae are a rich source of protein, with microalgae containing 20–60% and macroalgae (or seaweed) 1.3–47% of dry weight [20,21]. Algal proteins are high in essential amino acids like arginine and glutamine, which help maintain healthy blood flow and support cell function [22]. Leucine is the predominant essential amino acid in many seaweeds [23], while glutamic acid contributes to their umami taste [24]. Algae also contain branched-chain amino acids (valine, leucine, and isoleucine) in amounts similar to those found in soy and eggs, making them a valuable protein source for muscle health [1]. Many algal species are classified as complete proteins, providing all essential amino acids in amounts that meet the standards set by the FAO and WHO [25]. The amino acid composition of various microalgae and macroalgae species compared to conventional proteins (eggs and soybean) is described in detail by [1].

Evaluating protein quality helps determine the nutritional value of a food source. The most common methods include the Amino Acid Score and the Protein Digestibility-Corrected Amino Acid Score (PDCAAS), both recommended by the FAO and WHO [26]. AAS determines the nutritional quality of a protein compared to a reference protein. Higher scores reflect better nutritional quality and bioavailability [26]. Animal proteins generally score highest due to their balanced amino acid profile, with casein ranging from 1.03 to 1.32 [27,28]. Microalgae like *C. vulgaris* and *C. sorokiniana* (Chlorophyta) have AAS values of 1.10 and 1.16, respectively, which are close to casein [29]. PDCAAS measures protein quality by assessing amino acid composition and digestibility. Microalgae such as *T. obliquus*, *C. vulgaris*, *C. sorokiniana*, and *L. platensis* have PDCAAS values between 0.63–0.84. While these are lower than scores for animal proteins, they are comparable to plant proteins. The lower scores are mainly due to reduced digestibility caused by algae’s tough cell walls, highlighting the importance of improving processing methods to increase their nutritional value [29–31].

### 1.3. Essential nutrients and bioactive compounds

In recent years, microalgae have emerged as a promising resource in academic, industrial, and commercial sectors due to their ability to produce a diverse array of high-value compounds [32]. These include renewable biofuels (such as biogas, biodiesel, and bioethanol), bioactive molecules (including carotenoids, lipids, peptides, lutein, and astaxanthin), and a wide spectrum of essential vitamins such as A, C, E, and B-complex, as well as polyunsaturated fatty acids (PUFAs). Additionally, microalgae serve as sources of pigments, antioxidants, and compounds with applications in nutraceuticals, pharmaceuticals, and cosmetics. The growing interest in microalgal products reflects their vast potential in sustainable energy, human health, and functional food innovation [11]. Table 1 provides an overview of the key bioactive compounds derived from various microalgal species.

**Table 1.** Micro-algal bioactive compounds and their applications.

Bioactive compounds	Source	Applications
Phycobiliproteins, Phycocyanin, *Porphyridium*, phycoerythrin and chlorophyll A protein	Cyanophyceae, Rhodophyta, *Dolichospermum flos-aquae* (formerly *Anabaena flos-aquae*) (Cyanophyceae), *Caulerpa racemosa* (Chlorophyta), *Spirulina/Limnospira*, *Chlorella* sp., *Caulerpa racemosa* (Chlorophyta), *Porphyridium* (Rhodophyta), *Scenedesmus/Tetradesmus* (Chlorophyta), *Nitzschia incerta* (Bacillariophyceae)*, Microcystis aeruginosa* (Cyanophyceae), Cryptophyceae, *Dolichospermum flos-aquae* (Cyanophyceae)	Antioxidant, anti-inflammatory, immune activators, cytotoxic to tumor cells, prevent atherosclerosis, coronary diseases and cancer. Functional foods, used in animal feed supplements, bioplastics production, and photo-ageing protective formulations. Food antioxidants, food coloring agents, humans and plants
Polysaccharides (Starch, hemicellulose, cellulose, pectin)	*Chlorella vulgaris* (Chlorophyta), *Margalefidinium polykrikoides* (Dinophyceae), Fucus vesiculosus (a marine brown macroalga)	Nanocellulose, cosmeceuticals, biofilters, bioplastics, biofuels
Sulphated polysaccharides (carrageenan, GAG, agar, ulvans, lectin, galactan, naviculan, fucoidans, alginate, laminaran, Stypodiol, isoepitaondiol, taondiol)	Marine microalgae *Sargassum wightii* (Phaeophyceae), *Porphyridium* sp.*, Turbinaria conoides* (Phaeophyceae), *Porphyra/Pyropia* sp. (Rhodophyta)	Antioxidative, anti-inflammatory and immunomodulatory properties. Preventive and curative agents in viral infections.
Lipids MUFAs and PUFAs (Arachidonic acid, EPA, DHA)	*Spirulina/Limnospira* (Cyanophyceae), *Porphyridium* sp. (Rhodophyta), *Crypthecodinium cohnii* (Dinophyceae), *Scenedesmus/Tetradesmus* (Chlorophyta), *Lobosphaera incisa* (Chlorophyta), *Nannochloropsis* sp. (Eustigmatophyceae), *Phaeodactylum tricornutum* (Bacillariophyceae), *Schizochytrium* sp., *Ulkenia* sp. (Labyrinthulomycetes)	Cardiovascular benefits, anti-inflammatory, mental development, improves brain function, vision and functional development, improve infants’ growth,
Vitamins (A, C, E, B1, B6, B12, biotin, riboflavin, nicotinic acid, pantothenate, folic acid)	*Spirulina/Limnospira* sp. (Cyanophyceae), *Dunaliella tertiolecta* (Chlorophyta), *Chlamydomonas* sp*., Chlorella* sp. (Chlorophyta), *Chaetoceros calcitrans* (Mediophyceae), *Scenedesmus*/*Tetradesmus* sp. (Chlorophyta), *Nannochloropsis oculata* (Eustigmatophyceae), *Prototheca zopfii*, *T. suecica* (Chlorophyta)	Antioxidants, Food supplements and Nutraceuticals, reduce breast cancer risk, DNA repair and chemo-preventive activities, cosmetics
Phenolic acids caffeic acids, chlorogenic acids	*Chlorella vulgaris* (Chlorophyta), *Nannochloropsis* sp. (Eustigmatophyceae), *Isochrysis* sp. (Coccolithophyceae)	Possess anticancer, antispasmodic and antioxidant properties

Source: (33).

## 2. Search strategy and study selection criteria

This review explores the potential of algal peptides in health promotion, with a focus on bioactive compounds derived from both macroalgae and microalgae. A thorough search was conducted across a variety of scientific databases, including Google Scholar (http://www.scholar.google.co.in), PubMed (http://www.ncbi.nlm.nih.gov/pubmed), Elsevier (https://www.elsevier.com/en-in), Science Direct (http://www.sciencedirect.com), Springer (http://www.springer.co.in), and Scopus (http://www.scopus.com). Studies were selected based on their inclusion of bioactive peptides that have been scientifically validated for their significant impact on molecular mechanisms related to health promotion and disease prevention. Priority was given to studies published in respected, peer-reviewed journals to ensure the credibility and reliability of the findings. Emphasis was placed on well-documented peptides from both macroalgae and microalgae that have demonstrated their effectiveness in promoting health.

## 3. Health benefits of algal proteins

**Figure 1.**
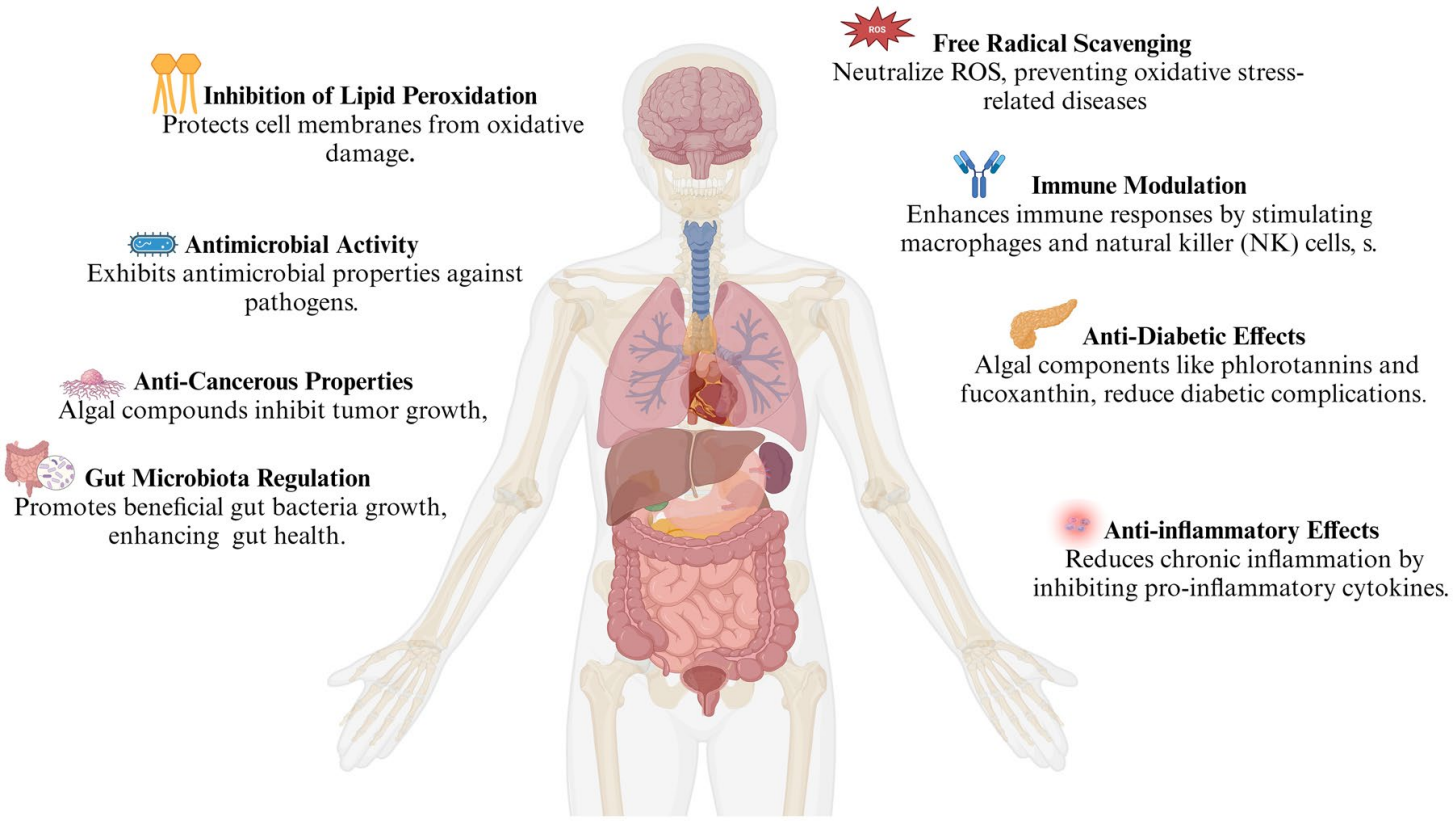
Health benefits of algal peptides. This diagram illustrates the key biological effects of algal-derived bioactive compounds, including antioxidant, antimicrobial, anticancer, antidiabetic, and anti-inflammatory actions, as well as gut microbiota modulation, contributing to enhanced immune function, metabolic health, and disease prevention.

The global rise in non-communicable diseases (NCDs), including obesity, cardiovascular disorders, type 2 diabetes, and hypertension, demands innovative and integrative therapeutic strategies [34]. These chronic conditions are closely interconnected through shared pathological mechanisms such as persistent low-grade inflammation, oxidative stress, and metabolic dysfunction [35]. Among these, inflammation plays a central role by contributing to insulin resistance, endothelial dysfunction, lipid accumulation, and vascular injury, thereby accelerating disease progression [36,37]. Obesity further amplifies systemic inflammation, creating a self-perpetuating cycle that markedly increases the risk of cardiovascular disease and diabetes [38]. Given these complex interactions, interventions targeting inflammatory and metabolic dysregulation hold promise for addressing multiple NCDs simultaneously. In this context, this review explores the health-promoting potential of algal-derived peptides, with a focus on their ability to improve individual disease outcomes while modulating shared pathological pathways, as summarized in Figure 1.

### 3.1. Impact on hypertension

Hypertension, widely known as the “silent killer,” is a chronic, multifactorial disease and a leading global health concern [39]. It plays a pivotal role in the onset and progression of cardiovascular diseases (CVD), and if not properly managed, can lead to serious complications such as heart attacks, strokes, and kidney failure [40–42]. Hypertension arises from a complex interplay of factors such as obesity, insulin resistance, poor diet, stress, aging, and inactivity. These diverse etiological components emphasize the necessity for integrated, evidence-based prevention and management strategies to mitigate its progression and associated health risks [43].

Blood pressure regulation primarily involves the renin-angiotensin system (RAS), where the enzymes renin and angiotensin-converting enzyme ACE play central roles [44]. Renin converts angiotensinogen into angiotensin I, which is then transformed by ACE into angiotensin II, a potent vasoconstrictor that raises blood pressure. Excessive activity of renin or ACE leads to high levels of angiotensin II, causing abnormal vessel constriction and reduced vasodilation [45]. Additionally, ACE degrades bradykinin, a peptide that promotes vessel relaxation, further contributing to hypertension [46]. A primary therapeutic approach involves using ACE inhibitors to block the formation of angiotensin II, encouraging vasodilation and lowering blood pressure [47]. Another crucial mechanism involves nitric oxide (NO) production by endothelial nitric oxide synthase (eNOS); NO induces vasodilation, and its deficiency under high angiotensin II levels leads to increased constriction of blood vessels [48].

**Figure 2.**
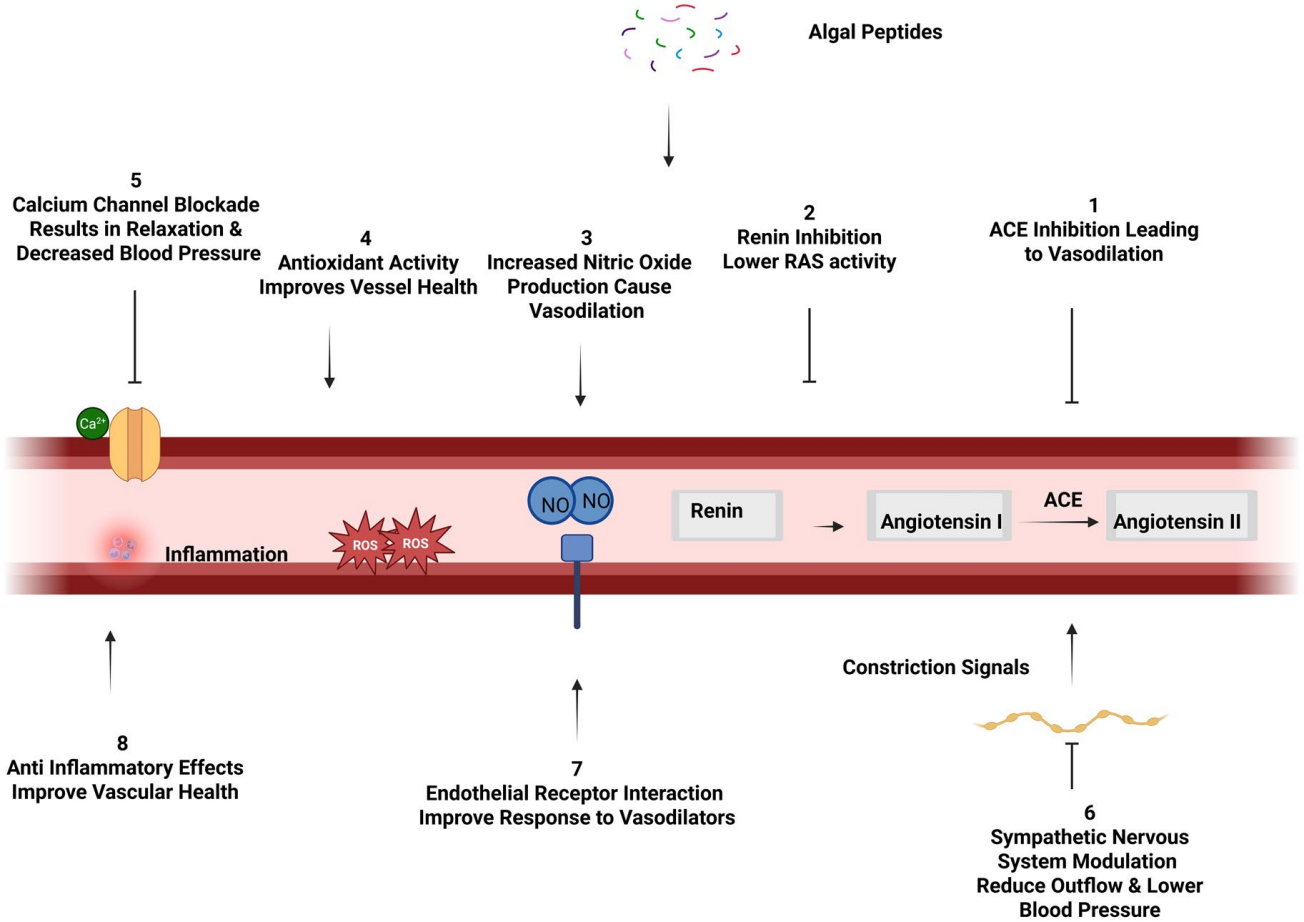
Mechanisms of antihypertensive action of algal peptides. This diagram illustrates the proposed antihypertensive mechanisms of algal peptides, including ACE and renin inhibition, nitric oxide production, antioxidant and anti-inflammatory effects, and modulation of vascular and neural pathways, collectively contributing to improved vascular function and reduced blood pressure.

Additional mechanisms involved in blood pressure regulation include the sympathetic nervous system, which controls heart rate and blood vessel tone, and antioxidant defenses that protect blood vessels from oxidative stress [49,50]. Maintaining healthy endothelial function is also critical, as it enhances the blood vessels’ ability to respond to vasodilators and regulate vascular tone effectively [51]. Moreover, controlling inflammation by reducing pro-inflammatory cytokines and blocking inflammatory pathways helps preserve vascular health and prevent hypertension [52]. Figure 2 illustrates how algal peptides support antihypertensive effects through multiple mechanisms regulating vascular function and blood pressure.

Among seaweed-derived peptides, species such as *Undaria pinnatifida* (Phaeophyceae), *Neopyropia yezoensis* (formerly *Porphyra yezoensis*) (Rhodophyta), *Ulva rigida*, and *Ulva clathrata* (formerly *Enteromorpha clathrata*) have shown ACE inhibitory activity [53–57]. In particular, *Palmaria palmata* (Rhodophyta) yields renin-inhibitory peptides with notable bioactivity, including synthetic analogs like IRLIIVLMPILMA. Other promising peptides have been identified from *Gracilariopsis lemaneiformis*, *Mazzaella japonica* (Rhodophyta), and *Ulva intestinalis* (Chlorophyta), each contributing to ACE inhibition and cardiovascular health [58–60].

On the microalgal front, species such as *Limnospira platensis* (formerly *Spirulina platensis*) (Cyanophyceae), *Chlorella vulgaris* (Chlorophyta), *Auxenochlorella pyrenoidosa* (formerly *Chlorella pyrenoidosa*), *Chlorella sorokiniana* (Chlorophyta), *Nannochloropsis oculata* (Eustigmatophyceae), *Tetradesmus obliquus* (Chlorophyta), and *Isochrysis galbana* (Coccolithophyceae) have emerged as rich sources of antihypertensive peptides [44,61–66]. Peptides such as VECYGPNRPQF from *Chlorella vulgaris* [44] and tripeptides like Trp-Val and Val-Trp from *Chlorella sorokiniana* [62] exhibit potent ACE inhibition. Similarly, peptides from *Dunaliella salina* (Chlorophyta) (e.g. ILTKAAIEGK, IIYFQGK) and *Nannochloropsis oculata* (GMNNLTP, LEQ) have demonstrated antioxidant and ACE inhibitory effects relevant to blood pressure regulation, respectively [63,67].

Beyond direct inhibition of ACE or renin, algal peptides exert complementary vascular benefits. For instance, a novel peptide (GIVAGDVTPI) derived from *Limnospira platensis* was shown to promote endothelial nitric oxide (NO) production *via* activation of the PI3K/AKT pathway, leading to vasorelaxation and improved serum nitrite levels *in vivo* [68]. Similar vasodilatory and antioxidant effects have been attributed to peptides from *Chlorella vulgaris* and *Palmaria palmata* (Rhodophyta), contributing to vascular homeostasis through both NO modulation and reactive oxygen species (ROS) scavenging [69].

Additional bioactive compounds from algae, such as pigments and polysaccharides, also enhance cardiovascular health. Phycocyanin, a pigment-protein complex from *Limnospira*, has demonstrated antioxidant properties that reduce oxidative stress and improve endothelial function, thereby enhancing the antihypertensive effects of co-occurring peptides [70]. Furthermore, polysaccharides from brown seaweeds like *Saccharina japonica* (formerly *Laminaria japonica*) (Phaeophyceae) have been shown to lower blood pressure through modulation of gut microbiota and stimulation of short-chain fatty acid (SCFA) production, which collectively improve vascular integrity and reduce systemic inflammation [1].

Algal peptides are also increasingly recognized for their anti-inflammatory capabilities. They help attenuate vascular inflammation by downregulating pro-inflammatory cytokines and inhibiting key inflammatory signaling pathways [71]. Notably, peptides from *Limnospira platensis* have been shown to suppress inflammatory marker expression in endothelial cells, promoting a healthier vascular environment [72]. By reducing inflammation, these peptides play a supportive role in restoring endothelial function and managing hypertension. This natural anti-inflammatory potential has led to the formal recognition of certain algae-based products as functional foods [73].

Importantly, evidence from human intervention studies supports the translational relevance of these antihypertensive effects. Randomized and placebo-controlled trials have demonstrated that supplementation with algal products such as *Limnospira* (*Spirulina*) and *Chlorella* leads to significant reductions in systolic and diastolic blood pressure in individuals with mild to moderate hypertension [74–76]. Daily intake of *Spirulina* (2–4.5 g/day) over periods ranging from 8 to 12 weeks has consistently been associated with improvements in systolic blood pressure, both as a standalone supplement and when incorporated into functional foods, whereas no comparable effects were observed in placebo groups [74–76]. Similarly, long-term supplementation with *Chlorella* (approximately 1.5 g/day for up to six months) has been shown to reduce blood pressure in hypertensive and borderline hypertensive adults, with enhanced effects reported for GABA-enriched formulations [77,78]. Beyond microalgae, macroalgal preparations have yielded comparable outcomes; consumption of dried wakame powder significantly lowered systolic and diastolic blood pressure in older hypertensive adults [79], while enzymatically derived nori peptides reduced blood pressure in hypertensive patients without affecting normotensive individuals [56]. Collectively, these findings indicate that algal-derived peptides and algal-based functional foods exert clinically meaningful antihypertensive effects in humans, supporting their role as safe and effective complementary dietary strategies for blood pressure management. It should be noted that most human studies employed whole algal biomass or extracts, and the specific contribution of individual peptides remains to be fully elucidated.

Notably, the Ministry of Health, Labour and Welfare of Japan has approved enzymatic digests of *Neopyropia yezoensis* (formerly *Pyropia yezoensis*) (Rhodophyta) and *Undaria pinnatifida* (Phaeophyceae) (commonly known as wakame) as Foods for Specified Health Uses (FOSHU), based on their demonstrated antihypertensive effects [71] along with reported IC_50_ values that demonstrate their potent enzyme-inhibitory activity. Table 2 provides an overview of major algal-derived bioactive peptides, their respective sources, and their antihypertensive mechanisms, along with reported IC_50_ values that demonstrate their potent enzyme-inhibitory activity. The table also highlights evidence from randomized controlled trials and placebo-controlled human interventions evaluating these peptides or algal supplements, underscoring their therapeutic promise in blood pressure reduction and vascular protection.

**Table 2.** Anti-hypertensive effects of algal peptides.

Peptides sequence	Peptide sources	IC_50_ values	Type of study	Mechanism	References
*Chlorella* peptide	*Chlorella* (Chlorophyta)	–	*in vivo* (rat trial)	Modulation of RAS	[80]
Thr-Met-Glu, Pro-Gly-Lys-Pro	Spirulina/Limnospira *sp.*	0.98 ± 0.02 mg/ml	*in vitro*	ACE inhibition	[61]
YH KY FY IY	*Undaria pinnatifida* (Phaeophyceae)	5.1 μM 7.7 μM 3.7 μM 2.7 μM	*in vitro*	ACE inhibition	[57]
IY AKYSY MKY LRY	*Neopyropia yezoensis* (formerly *Porphyra yezoensis*) (Rhodophyta)	2.96 μM 1.52 μM 7.26 μM 5.06 μM	*in vivo* (human trial)	Decreases blood pressure	[56]
*PAFG [*11] (Pro-Ala-Phe-Gly)	*Ulva clathrate* (formerly *Enteromorpha clathrata*) (Chlorophyta)	35.9 μM	*in vitro*	ACE inhibition	[57]
*Chlorella* hydrolysates	*Auxenochlorella pyrenoidosa* (formerly *Chlorella pyrenoidosa*) (Chlorophyta)		*in vivo* (rat trial)	Inhibited ACE activity	[66]
VECYGPNRPQF	*Chlorella vulgaris* (Chlorophyta)	0.91 ± 0.31 μM	*in vitro*	ACE inhibition, NO production, Antioxidant	[44]
TGAPCR, FQIN[M(O)]CILR	*Gracilariopsis lemaneiformis* (Rhodophyta)	23.94 ± 0.82 μM 9.64 ± 0.36 μM	*in vivo* (rat trial)	ACE inhibition; Antioxidant	[59]
Ile-Arg-Leu-Ile-Ile-Val-Leu-Met-Pro-Ile-Leu- Met-Ala (SDITRPGGQM, IRLIIVLMPILMA)	*Palmaria palmata* (Rhodophyta)	Fraction 25 (Fr-25) inhibited renin activities by 58.97 % (±1.26) at a concentration of 1 mg/mL	*in vitro*	Antioxidant; Renin inhibition; NO production	[55,81]
Gly-Met-Asn-Asn-Leu-Thr-Pro (GMNNLTP), Leu-Glu-Gln (LEQ)	*Nannochloropsis oculata* (Eustigmatophyceae)	123 μM 173 μM	*in vitro*	ACE inhibition	[63]
ILTKAAIEGK, IIYFQGK, NDPSTVK, TVRPPQR	*Dunaliella salina* (Chlorophyta)	–	*in vitro*	ACE inhibition, Antioxidant	[67]
WV, WYGPDRPKFL	*Tetradesmus obliquus* (Chlorophyta)	5.73 and 0.82 μmol *L* −1 5.73 and 0.82 μmol *L* −1 57.3 μmol/L 0.82 μmol/L	*in vitro*	ACE inhibition	[64]
YMGLDLK	*Isochrysis galbana* formerly *Polysiphonia urceolata*) (Rhodophyta)	36.1 μM	*in vitro*	ACE inhibition	[65]
YRD, VSEGLD, TIMPHPR, GGPAT, SSNDYPI, SRIYNVKSNG, VDAHY, CPYDWV, YGDPDHY, NLGN, DFGVPGHEP	*Mazzaella japonica* (Rhodophyta)	–	*in vitro*	ACE inhibition	[58]
IDHY, LVVER	*Gracilariopsis chorda* (Rhodophyta)	–	*in vitro*	ACE inhibition	[82]
ARY, YLR, LRM	*Pyropia pseudolinearis* (Rhodophyta)	1.3 μmol 5.8 μmol 0.15 μmol	*in vitro*	ACE inhibition	[83]
LLGRC, FLKPLGSGK, MSANHDAGGS, LLSKT, LLTKS	*Auxenochlorella pyrenoidosa* (formerly *Chlorella pyrenoidosa*) (Chlorophyta)	ACE inhibition reported as 84.2% at 1 mg/mL	*in vitro*	ACE inhibition	[84]
FDGIP (FP-5), AIDPVRA (AA-7)	*Caulerpa lentillifera* (Chlorophyta)	58.89 ± 0.68 μM 65.76 ± 0.92 μM	*in vitro*	ACE inhibition	[85]
Trp-Val, Val-Trp, Ile-Trp, Leu-Trp	*Chlorella sorokiniana* (Chlorophyta)	307.61 ± 0.01 μM 0.58 ± 0.02 μM 0.50 ± 0.01 μM 1.11 ± 0.02 μM	*in vitro*	ACE inhibition	[62]
Phe-Gly-Met-Pro-Leu-Asp-Arg (FGMPLDR), Met-Glu-Leu-Val-Leu-Arg (MELVLR)	*Ulva intestinalis* (Chlorophyta)	219.35 μM 236.85 μM	*in vitro*	ACE inhibition	[60]
AFL (Ala-Phe-Leu), IP (Ile-Pro)	*Ulva rigida* (Chlorophyta)	65.9 μM 87.6 μM	*in vitro*	ACE inhibition	[54]
ALLAGDPSVLEDR, VVGGTGPVDEWGIGAR	Bangia fuscopurpurea (Rhodophyta)	57.2 ± 5.0 μg/mL 66.2 ± 4.2 μg/mL	*in vitro*	ACE inhibition	[86]
Clinical Trials and RCT					
Study/Population	Algal Source	Dose	Duration	Key findings	Reference
Hypertensive adults (RCT) *n* = 40	*Spirulina* (*Limnospira maxima*)	2 g/day	3 months	Significant reduction in systolic BP vs. placebo	[74]
Patients on ACE-inhibitor therapy (pilot trial) *n* = 16	*Spirulina/Limnospira*	4.5 g/day	12 weeks	Reduction in systolic BP compared with baseline	[75].
Adults with elevated BP (functional food trial) *n* = 41	*Spirulina/Limnospira platensis* (salad dressing)	2 g/day	8 weeks	Significant decreases in systolic & diastolic BP vs. placebo	[76]
Borderline hypertensive adults (double-blind)	GABA-rich *Chlorella*	GABA-enriched dose (not specified)	12 weeks	Significant reduction in systolic BP	[**78]**
Older hypertensive adults (case-controlled) *n* = 36	Wakame (*Undaria pinnatifida*)	Dried wakame powder	8 weeks	Significant reductions in systolic & diastolic BP vs. controls	[79]
Spontaneously hypertensive rats (preclinical)	Nori-peptide AKYSY (*Neopyropia yezoensis*)	0.2 mg/kg (peptide); 200 mg/kg	Acute & long-term	Significant BP reduction and decrease in ACE activity	[56]
Hypertensive adults (*n* = 64)	Nori-peptides (*Neopyropia yezoensis*)	1.8 g/day	35 days	Significant reductions in systolic & diastolic BP; safe with no adverse events	[56]

### 3.2. Potential role in diabetes

Diabetes mellitus is a metabolic condition characterized by persistent hyperglycemia, mostly caused by decreased insulin secretion or activity. Approximately 830 million people globally have diabetes, primarily in low- and middle-income countries [87]. While pharmaceutical therapies are essential, effective diabetes management also includes dietary strategies. For example, consuming meals with a low glycemic index (GI), which helps to slow down digestion and absorption and improve glycemic control [88].

A common antidiabetic strategy involves inhibiting α-amylase and α-glucosidase, enzymes that break down complex carbohydrates into glucose, thus delaying starch breakdown, glucose absorption and reducing postprandial blood glucose spikes [89]. Several algal peptides have demonstrated strong inhibitory effects on these enzymes. For example, peptides from *Limnospira platensis* [90], *Ulva lactuca* [91], *Dunaliella salina* [92], and *Gracilaria bursa-pastoris* (Rhodophyta) [93] have been shown to reduce enzyme activity and consequently lower blood glucose levels. In a study by Hu et al. [94], three synthesized peptides from *Limnospira platensis*, GVPMPNK, RNPFVFAPTLLTVAAR, and LRSELAAWSR, were evaluated, with LRSELAAWSR showing moderate inhibition of α-amylase and strong inhibition of α-glucosidase. Two novel peptides, Gly-Gly-Ser-Lys and Glu-Leu-Ser, derived from enzymatic hydrolysates of red seaweed laver (*Porphyra/Pyropia/Neopyropia* sp.), showed strong α-amylase inhibition with IC_50_ values of 2.58 ± 0.08 mM and 2.62 ± 0.05 mM, respectively [68].

DPP-IV is an enzyme that degrades incretin hormones like GLP-1, which play a crucial role in enhancing insulin secretion post-meal. By inhibiting DPP-IV, algal peptides prolong incretin activity, thereby improving insulin release and glucose homeostasis. Peptides from *Chlorella sorokiniana* (e.g. Val-Trp, Trp-Val, Ile-Trp, Leu-Trp) demonstrated DPP-IV inhibition along with GLUT-4 upregulation, enhancing glucose uptake in cells [95]. Similarly, *Limnospira platensis* peptide LRSELAAWSR exhibited potent DPP-IV inhibition (IC_50_ = 167.3 μg/mL) [94]. Computational docking studies have also highlighted the DPP-IV inhibitory potential of metabolites and proteins from *Caulerpa* sp. (Chlorophyta), suggesting strong binding affinity and anti-diabetic activity, though further *in vitro* and *in vivo* studies are required [96]. Additionally, simulated digestion of *Palmaria palmata* protein hydrolysates also exhibited DPP-IV inhibitory activity [97]. Peptides obtained from *Limnospira platensis* have also shown their DPP-IV inhibitory potential [90].

Algal peptides support insulin secretion by stimulating pancreatic β-cell activity and incretin modulation. *Palmaria palmata* protein hydrolysates elicited significant insulinotropic effects (*p* < 0.05–0.001), and their simulated digestion retained or even enhanced insulin secretion and GIP release [97]. Similarly, supplementation with *Saccharina japonica* (sea tangle) improved insulin secretion over 13 weeks in streptozotocin (STZ)-induced diabetic rats [98]. *Ecklonia cava* (Phaeophyceae) also exhibited insulin *via* activation of AMP-activated protein kinase (AMPK)/ACC and PI3K/Akt signaling pathways [99], reinforcing its therapeutic potential.

Chronic hyperglycemia leads to oxidative stress and inflammation, contributing to β-cell damage and insulin resistance. Algal peptides have demonstrated strong antioxidant properties that alleviate oxidative stress associated with diabetes. For example, *Gracilariopsis lemaneiformis* peptides have good antioxidant potential [100]. According to Kaur et al. [101], these peptides help preserve pancreatic function and reduce oxidative burden. Inflammation also plays a key role in insulin resistance. Algal peptides have been shown to downregulate pro-inflammatory cytokines like TNF-α and IL-6, thereby improving insulin sensitivity [102].

Dyslipidemia often accompanies diabetes, increasing the risk of cardiovascular complications. Algal peptides have been found to positively modulate lipid profiles. Studies on *Limnospira platensis* and *Chlorella vulgaris* in animal models revealed reductions in total cholesterol, LDL, and triglycerides [103,104]. Furthermore, *Caulerpa racemosa* (Chlorophyta) extract significantly lowered blood glucose levels in diabetic rats at both 100 mg/kg and 200 mg/kg doses, further supporting its metabolic benefits [105].

The proteins obtained from *Undaria pinnatifida* have high nutritional content and bioactivity. The bioactive ingredients obtained help in reducing blood glucose levels and help in diabetes management [106]. Oral administration of *Nannochloropsis oculata* significantly reduced serum glucose and increased insulin levels in diabetic rats [107]. Table 3 shows that algal peptides have diverse mechanisms, including enzyme inhibition (α-amylase, α-glucosidase, DPP-IV), incretin enhancement, insulin stimulation, and antioxidant/anti-inflammatory effects, making them promising candidates for the development of functional foods or nutraceuticals in diabetes management.

**Table 3.** Anti-diabetic effects of algal peptides.

Algal source	model	Peptides	Effects	References
*Spirulina platensis*	*in vitro*	*Limnospira platensis* peptides	Inhibits α-amylase, α-glucosidase, and DPP-IV; improves insulin sensitivity; increases GLUT-4 expression	[103]
*Spirulina platensis*	*in vitro*	GVPMPNK, RNPFVFAPTLLTVAAR, and LRSELAAWSR)	Inhibits α-amylase, α-glucosidase, and DPP-IV	[94]
*Limnospira platensis* formerly *Arthrospira platensis*)	*in vitro*	*Limnospira platensis* peptides	Inhibits α-amylase, α-glucosidase, and DPP-IV	[90]
*Porphyra* sp.	*in vitro* + animal glucose models	Gly-Gly-Ser-Lys and Glu-Leu-Ser	Inhibits α-amylase IC_50_ value: 2.58 ± 0.08 mM (Gly-Gly-Ser-Lys) and 2.62 ± 0.05 mM (Glu-Leu-Ser).	[68]
*Chlorella sorokiniana* (Chlorophyta) (PCH)	*in vitro*	Val-Trp, Trp-Val, Ile-Trp, and Leu-Trp	Inhibits α-glucosidase and DPP-IV; lowers plasma glucose	[95]
*Palmaria palmata* (Rhodophyta)	*in vitro*	Palmaria palmata peptides	Increases insulin and GLP-1; reduces blood glucose and HbA1c levels	[97]
*Ulva lactuca* (Chlorophyta)	*in vitro*	*Ulva lactuca proteins*	Anti-diabetic efficacy *in vitro*	[91]
*Gracilaria bursa-pastoris* (Rhodophyta)	*in vitro*	*G. bursa-pastoris* extracts	Exhibits *in vitro* anti-diabetic properties	[93]
*Dunaliella salina* (Chlorophyta)	*in vitro*	*Dunaliella salina* protein	Acts as an amylase inhibitor; functional food potential for diabetes	[92]
*Caulerpa* sp.	*in vitro* and *in vivo*	*Caulerpa* sp. proteins	DPP-4 inhibitors identified through metabolite screening	[96]
*Caulerpa racemosa* (Chlorophyta)	*in vitro*	*C.rosemosa* extract	Decreased blood glucose level	[108]
*Caulerpa racemosa* (Chlorophyta) *(Sea grape)*	Diabetic rats	*C.rosemosa* extract	Lowers blood glucose; contains antioxidant and antidiabetic phytochemicals	[105]
*Saccharina japonica* (formerly *Laminaria japonica*) *(Sea Tangle)*	STZ induced diabetic rats	*Laminaria japonica* powder extract	Improves blood glucose	[98]
*Ecklonia cava* (Phaeophyceae)	Type 1 diabetic rats	*Ecklonia cava* peptides	Activates AMPK and Akt pathways in type 1 diabetes models	[99]
*Nannochloropsis* sp.	Diabetic rats	*Nannochloropsis* proteins	Promotes GLUT-4 mediated glucose uptake; lowers blood glucose	[107]

**Figure 3.**
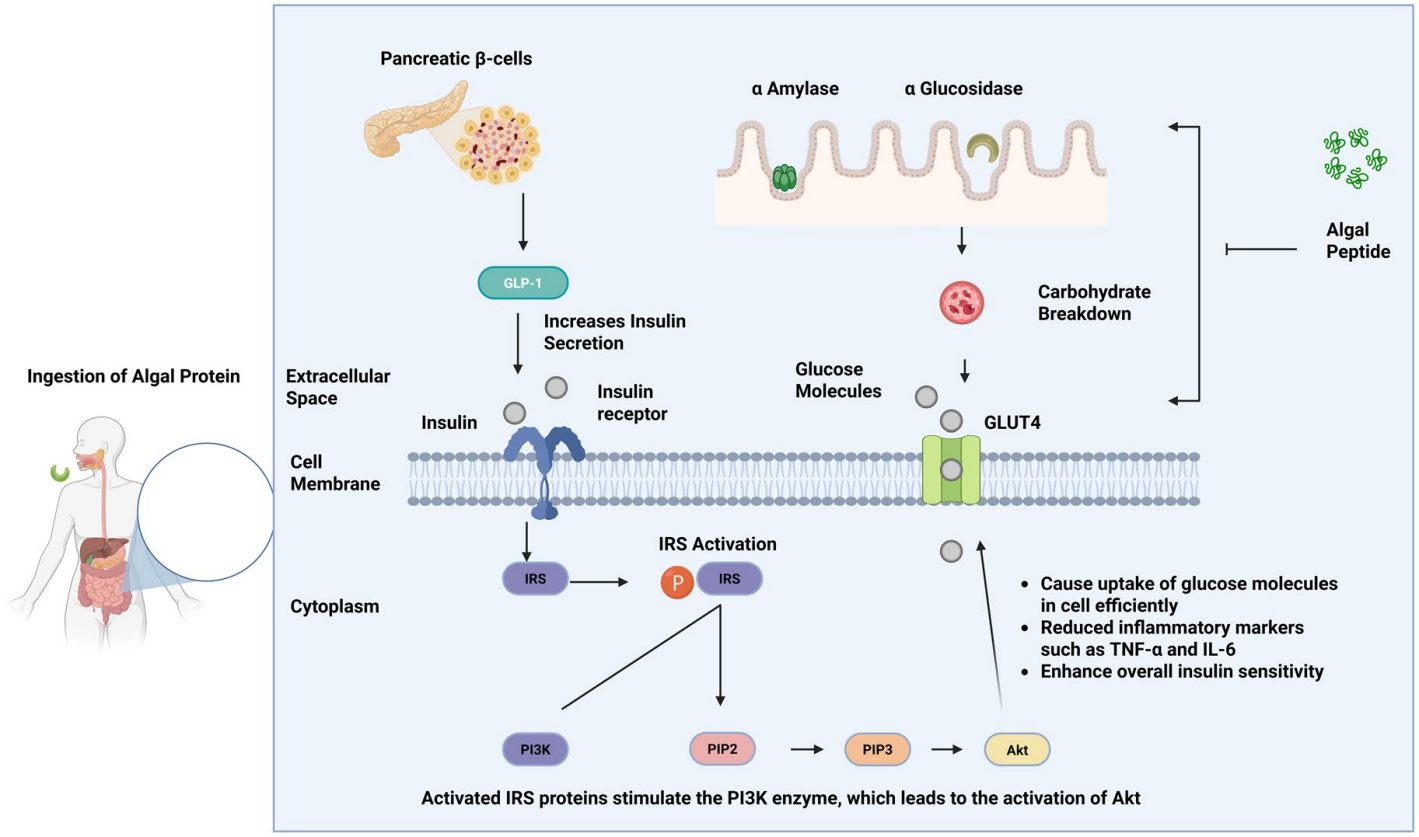
Mechanism of anti-diabetic effects of algal peptides. The diagram illustrates how algal-derived peptides regulate blood glucose levels through multiple pathways. These peptides inhibit α-amylase and α-glucosidase, slowing carbohydrate digestion and glucose absorption. They also stimulate GLP-1 secretion, enhancing insulin release from pancreatic β-cells. Once insulin binds to its receptor, the IRS/PI3K/Akt signaling cascade is activated, leading to GLUT-4 translocation and increased cellular glucose uptake. Additionally, algal peptides reduce inflammatory cytokines like TNF-α and IL-6, improving insulin sensitivity. Collectively, these mechanisms support their potential in managing diabete.

Notably, evidence from human intervention studies supports the translational relevance of these antidiabetic effects. Clinical trials involving *Limnospira platensis* (*Spirulina*) supplementation in individuals with type 2 diabetes have consistently reported significant reductions in fasting blood glucose, improvements in glycated hemoglobin (HbA1c), and favorable modulation of lipid profiles, including reductions in total cholesterol, LDL cholesterol, and triglycerides [108]. These metabolic improvements align with the antioxidant, anti-inflammatory, and enzyme-inhibitory mechanisms observed in preclinical studies. Similarly, supplementation with *Chlorella vulgaris* has demonstrated clinically meaningful benefits, including reductions in fasting glucose levels, improvements in insulin sensitivity as reflected by decreased HOMA-IR values, and significant improvements in lipid parameters [109]. Some human trials have additionally reported reductions in oxidative stress biomarkers following *Chlorella* supplementation, supporting its role in mitigating diabetes-associated oxidative damage [109]. Although clinical studies specifically evaluating isolated macroalgal-derived peptides remain limited, the consistent findings from *Spirulina* and *Chlorella* trials provide compelling evidence that algal-derived bioactives can enhance glycemic regulation, lipid metabolism, and overall metabolic health in diabetic and pre-diabetic populations. Collectively, these findings support the potential application of algal-derived peptides and algal-based functional foods as complementary dietary strategies in diabetes management. However, direct evidence on the absorption, stability, and systemic availability of individual algal peptides in humans remains scarce. To confirm the therapeutic potential of algal peptides, well-designed clinical trials and human studies are required to validate the promising results seen in laboratory and animal models. Figure 3 illustrates algal-derived peptides regulate glucose metabolism through multiple mechanisms.

### 3.3. Cardioprotective effects

Cardiovascular disease represents the cumulative outcome of multiple interrelated risk factors, including hypertension, dyslipidemia, obesity, oxidative stress, endothelial dysfunction, and chronic low-grade inflammation. While the antihypertensive actions of algal-derived bioactive peptides have been discussed in the preceding section 3.1, growing evidence indicates that their cardiovascular benefits extend well beyond blood pressure control. Through coordinated effects on vascular function, lipid metabolism, redox balance, inflammatory signaling, and thrombosis-related pathways, algal peptides target several key processes involved in CVD initiation and progression. This section therefore focuses on the broader cardioprotective potential of algal-derived peptides, integrating mechanistic, preclinical, and clinical evidence.

Bioactive peptides derived from micro- and macroalgae exert multifaceted cardioprotective effects, as demonstrated in preclinical and *in vitro* studies. Several bioactive peptides isolated from algal species, including *Palmaria palmata* [110], *Gracilariopsis lemaneiformis* [59], *Caulerpa lentillifera* (Chlorophyta) [85], *Undaria pinnatifida* (Phaeophyceae)*, Ulva rigida* (Chlorophyta) [54], *Chlorella* [111], and *Spirulina/Limnospira* species [112], target the RAS and enhance nitric‐oxide production, contributing to systemic blood‐pressure reduction and vascular protection.

Obesity and dyslipidemia are closely linked risk factors that contribute to cardiovascular disease. Excess adipose tissue in obesity triggers pro-inflammatory cytokines (e.g. TNF-α, IL-6), which reduce endothelial nitric oxide and antioxidant capacity, leading to vascular damage. Dyslipidemia, characterized by elevated LDL-C and TGs levels, accelerates atherogenesis and plaque instability [113,114]. Apart from regulating hypertension to prevent CVD, algae exhibit anti-obesity properties, primarily through bioactive peptides that regulate lipid metabolism, inhibit adipogenesis, and enhance lipid oxidation pathways. A novel anti-lipidemic decapeptide PP1 (Leu-Leu-Val-Val-Trp-Pro-Trp-Thr-Gln-Arg) isolated from *Auxenochlorella pyrenoidosa* (formerly *Chlorella pyrenoidosa*) (Chlorophyta) *via* enzymatic hydrolysis inhibited porcine pancreatic lipase by 47.95% at 200 µg/mL, and in 3T3-L1 adipocytes reduced triacylglycerol accumulation by 27.9% at 600 µg/mL [115]. Multiple studies have demonstrated the hypolipidemic potential of *Limnospira platensis*-derived peptides. CANPHELPNK and NPVWKRK (3-5 kDa fraction) at 600 µg/mL inhibited pancreatic lipase by 72.7–88.1%, reduced 3T3-L1 preadipocyte proliferation by 32.3-60.1%, and decreased TGs accumulation by 19.5 and 23.7%, respectively. Preclinical trials on *Spirulina/Limnospira platensis* protein hydrolysate (SPPH) showed potential anti-obesity effects through modulation of the PPAR-γ, adipocytokine, AMPK, and MAPK signaling pathways. Similarly, Arg-Cys-Asp (IC_50_ = 6.9 µM) and Ser-Asn-Val (IC_50_ = 20.1 µM) peptides from fermented *L. platensis* inhibited HMG-CoA reductase, indicating strong hypocholesterolemic activity [116–118].

Several marine-algae-derived bioactive compounds show promising lipid-regulating and anti-obesity effects using diverse mechanisms. In silico proteolysis of *Ulva lactuca* RuBisCO predicted a suite of novel peptides with strong hypolipidemic potential, which set the stage for their targeted isolation and validation [119]. Moreover, *in vitro* assays of 1 kDa peptide permeate fractions from three macroalgae demonstrated potent pancreatic lipase inhibition, such as *P. palmata* peptides exhibiting an IC_50_ of 425.5 µg/mL, *U. lactuca* peptides 606.9 µg/mL, and *Alaria esculenta* peptides 653.3 µg/mL. Moreover, alcalase-generated hydrolysates from *A. esculenta* achieved 48% lipase inhibition at 5 mg/mL, and trypsin-derived peptides from *Ulva intestinalis* (MELVLR and FGMPLDR) showed IC_50_ values around 180 µg/mL, underscoring the broad promise of macroalgal peptides as natural agents to prevent obesity and dyslipidemia [120].

Algal-derived peptides also exert strong antioxidant effects that support cardiovascular health by protecting the vascular endothelium from oxidative stress and inflammation. They also enhance nitric oxide (NO) production, a critical molecule for maintaining vascular tone and promoting vasodilation, thus lowering atherosclerosis risk. Oxidative stress is a key metabolic contributor to the development and progression of cardiovascular diseases. Bioactive peptides from algae offer a multifaceted defense by neutralizing reactive oxygen/nitrogen species (ROS/RNS), chelating transition metals, and enhancing endogenous defenses by activating the Nrf2 pathway to upregulate antioxidant enzymes (SOD, GPx, HO-1, and CAT) [121]. Protein hydrolysates from *Auxenochlorella pyrenoidosa* demonstrated near-complete ABTS and DPPH radical scavenging at 5 mg/mL, along with lipid emulsion stabilization, highlighting both antioxidant capacity and food functionality [122]. Additionally, the VH12 peptide isolated from thioredoxin disulfide reductase of *Limnospira platensis* scavenged over 98% of DPPH radicals at 200 µM and reduced ROS levels by 55% in cardiac myocytes, without inducing cytotoxicity in human leukocytes [123].

Macroalgal peptides also have promising antioxidant effects, such as two peptides (ZD10 and ZD60) isolated from *Kappaphycopsis cottonii* (formerly *Eucheuma cottonii*) (Rhodophyta) protein hydrolysates, which scavenged ROS and protected human umbilical vein endothelial cells (HUVECs) from H_2_O_2_-induced apoptosis, indicating anti-atherosclerotic potential [124]. In red seaweed *Palmaria palmata*, peptide sequences obtained from pepsin and corolase hydrolysates exhibited significant antioxidant activity, with the SDITRPGGQM peptide showing the highest ferric-reducing antioxidant power (FRAP) and oxygen radical absorbance capacity (ORAC) [81]. Similarly, peptides isolated from *Ulva* species demonstrated strong antioxidant activity, with FRAP and ORAC values reaching 20 µmol Trolox equivalents per gram of protein [125].

Atherogenesis, the formation of atherosclerotic plaques within arterial walls, underlies major CVDs such as stroke, myocardial infarction, and coronary artery disease. Algal-derived peptides exhibit anti-inflammatory, antithrombotic, and anti-atherogenic properties. An undecapeptide (Val-Glu-Cys-Tyr-Gly-Pro-Asn-Arg-Pro-Gln-Phe) isolated from *Auxenochlorella pyrenoidosa* significantly downregulated endothelial adhesion molecules, including E-selectin, ICAM-1, and VCAM-1, as well as MCP-1 and endothelin-1, thereby suppressing atherogenic processes [126]. Similarly, peptides Leu-Asp-Ala-Val-Asn-Arg (P1) and Met-Met-Leu-Asp (P2) from *Limnospira maxima* hydrolysates inhibited P-selectin and E-selectin expression through suppression of Egr-1 *via* PKC/MAPK signaling pathways and histamine receptors. These peptides also reduced ROS levels and inhibited histamine and IL-8 release [127]. A current finding isolated a nonapeptide (Glu-Met-Phe-Gly-Thr-Ser-Ser-Glu-Thr) from *Isochrysis zhanjiangensis* (Coccolithophyceae) that reduced apoptosis and inflammation in ox-LDL-treated HUVECs, suggesting antiatherogenic potential, although its effects *in vivo* remain unconfirmed [128]. Moreover, recent *in vitro* screening of mixed microalgal hydrolysates identified novel low-molecular-weight peptides that inhibit cyclooxygenase (COX) activity with IC_50_ values comparable to NSAIDs, highlighting a direct enzyme-targeted mechanism for inflammation control [129]. Similarly, macroalgal peptides with 3–20 amino acid fragments from red and green seaweeds have been reported to block key inflammatory mediators, including COX-2 and inducible nitric oxide synthase, in endothelial and smooth muscle cells, thus reducing vascular adhesion molecule expression and monocyte recruitment to nascent plaques [54]. Indumathi et al. identified the anticoagulant properties of a peptide (NMEKGSSSVVSSRM) derived from a pepsin (EC 3.4.23.1)-treated hydrolysate *of Neopyropia yezoensis* [130].

Moreover, platelet-activating factor acetylhydrolase (PAF-AH), synthesized by macrophages, plays a key role in regulating inflammation by degrading platelet-activating factor (PAF). It is a potent mediator of thrombosis, angiogenesis, metastasis, and vascular disease. Elevated PAF-AH activity has been closely linked to atherosclerosis through promoting lipid oxidation and the accumulation of oxidized lipoproteins in blood vessels. Despite the development of synthetic inhibitors like Darapladib, clinical trials have shown limited success due to adverse effects [115]. As an alternative, Fitzgerald et al. identified NIGK (a tetrapeptide) obtained from papain hydrolysates of the seaweed *Palmaria palmata*, which has shown potential PAF-AH inhibitory activity with 2.32 mM IC_50_ values [131]. From microalgae, gastric-digested peptides P1 (LDAVNR) and P2 (MMLDF) derived from *L. maxima* have been reported to attenuate PAF-mediated inflammatory and adhesion responses, indicating their role in managing early atherosclerotic events [132]. Table 4 highlights the diverse cardiometabolic mechanisms through which algal-derived bioactive peptides contribute to cardiovascular health.

**Table 4.** Cardiometabolic mechanisms of algal-derived bioactive peptides.

Peptide sequence	Model	Species	Mechanism	References
Leu–Leu–Val–Val–Trp–Pro–Trp–Thr–Gln–Arg	*in vitro*	*Auxenochlorella pyrenoidosa* (Chlorophyta)	Pancreatic lipase inhibition ↓triacylglycerol accumulation	[115]
CANPHELPNK NPVWKRK	*in vitro*	*Limnospira platensis* (Cyanophyceae)	Pancreatic lipase inhibition ↓ 3T3-L1 proliferation ↓ TG accumulation (–19.5%)	[118]
Arg–Cys–Asp Ser–Asn–Val	*in vitro*	*Limnospira platensis* (Cyanophyceae)	HMG-CoA reductase inhibition	[116]
MELVLR FGMPLDR	*in vitro*	*Ulva intestinalis* (Chlorophyta)	Pancreatic lipase inhibition	[120]
Val–Glu–Cys–Tyr–Gly–Pro–Asn–Arg–Pro–Gln–Phe	*in vitro*	*Chlorella pyrenoidosa* (Chlorophyta)	↓E-selectin, ICAM-1, VCAM-1, MCP-1, ET-1 expression (anti-atherogenic)	[126]
Leu–Asp–Ala–Val–Asn–Arg (P1) Met–Met–Leu–Asp (P2)	*in vitro*	*Limnospira maxima* (Cyanophyceae)	↓ P-selectin & E-selectin *via* PKC/MAPK→Egr-1; ↓ ROS, IL-8, histamine (anti-inflammatory)	[127]
Glu–Met–Phe–Gly–Thr–Ser–Ser–Glu–Thr	*in vitro*	*Isochrysis zhanjiangensis* (Coccolithophyceae)	↓ apoptosis & inflammation in ox-LDL-treated HUVECs (anti-atherogenic)	[128]
NMEKGSSSVVSSRM	*in vitro*	*Neopyropia yezoensis* (Rhodophyta)	Anticoagulant properties	[130]
SDITRPGGQM	*in vitro*	*Palmaria palmate* (Rhodophyta)	Antioxidant activity High FRAP & ORAC values	[81]
VH12	*in vitro*	*Limnospira platensis* (Cyanophyceae)	Antioxidant, DPPH scavenging, ↓ ROS in cardiac myocytes	[123]
ZD10 ZD60	*in vitro*	*Kappaphycopsis cottonii* (Rhodophyta)	ROS scavenging; protection of HUVECs from H₂O₂-induced apoptosis	[124]
NIGK	*in vitro*	*Palmaria palmate* (Rhodophyta)	PAF-AH inhibition (anti-thrombotic/anti-inflammatory)	[131]
LDAVNR MMLDF	*in vitro*	*Limnospira maxima* (Cyanophyceae)	↓PAF-mediated inflammatory & adhesion responses (anti-atherosclerotic)	[132]

Importantly, emerging human intervention studies support the translational relevance of these cardioprotective effects. Clinical trials involving *Spirulina* supplementation in adults with hypertension and dyslipidemia have demonstrated significant reductions in systolic and diastolic blood pressure, total cholesterol, LDL-cholesterol, and triglycerides, alongside modest increases in HDL-cholesterol, indicating improvements in both hemodynamic and lipid-related cardiovascular risk factors [133]. Similarly, randomized controlled trials using *Chlorella* supplementation in hypercholesterolemic individuals have reported significant improvements in lipid profiles, including reductions in serum total cholesterol and LDL-cholesterol, together with favorable changes in endothelial function markers [134]. In addition to lipid modulation, intake of *Spirulina/Limnospira* and *Chlorella* has been associated with reduced oxidative stress biomarkers, such as malondialdehyde and reactive oxygen species, reflecting enhanced vascular antioxidant protection. Although clinical evidence remains limited in scale and duration, these findings provide encouraging support for the cardiovascular benefits of algal-derived bioactives and align closely with mechanistic and preclinical observations.

These findings suggest algae as natural, low-toxicity agents that present strong potential for integration into functional foods or nutraceutical formulations aimed at primary and adjunctive CVD prevention. Future studies should focus on clinical validation, optimal dosing strategies, and enhancing bioavailability to fully harness their cardiovascular benefits.

### 3.4. Potential role in weight management

Obesity is a multifaceted health issue influenced by genetic, environmental, and lifestyle factors [135]. Beyond the accumulation of excess body fat, it significantly increases the risk of developing metabolic disorders. At its core, obesity stems from an imbalance between calorie intake and energy expenditure. This imbalance is often exacerbated by poor dietary habits, sedentary lifestyles, and disruptions in metabolic signaling [136]. The regulation of energy homeostasis involves a complex network of hormonal and neuronal signals among the gastrointestinal tract, adipose tissue, pancreas, and hypothalamus. Key hormones, like insulin and adiponectin, play pivotal roles in modulating appetite, satiety, glucose metabolism, and fat storage [137]. In individuals with obesity, this regulatory system often malfunctions. Additionally, insulin resistance impairs the body’s ability to utilize glucose effectively, promoting fat accumulation [138,139]. Compounding these issues is chronic low-grade inflammation, characterized by elevated pro-inflammatory cytokines like TNF-α and IL-6, which further disrupt insulin signaling and lipid metabolism [38,140].

Algal peptides have emerged as promising natural agents due to their multifaceted effects on fat metabolism, energy regulation, and appetite control [141]. These compounds act through a variety of biochemical pathways, offering a holistic approach to weight management without the adverse effects often associated with synthetic drugs [101].

One of the exceptional ways algal peptides assist in weight management is by influencing the type of fat stored in the body. Our bodies contain different types of fat cells: white fat, which stores energy, and brown or beige fat, which burns energy to produce heat [142,143]. Several algal peptides have been shown to activate peroxisome proliferator-activated receptor gamma (PPAR-γ), a transcription factor central to adipocyte differentiation and lipid metabolism [144].

PPAR-γ activation stimulates a process called “browning” of white adipose tissue. This process transforms regular white fat cells into beige fat cells, which behave more like brown fat. Beige fat is metabolically active; it burns calories and releases energy as heat. This means that instead of storing excess calories, the body is encouraged to expend them, which can gradually lead to fat loss and better weight control [145]. Peptides derived from the brown seaweed *Undaria pinnatifida* and the Cyanophyceae *Limnospira platensis* (*Spirulina*) have shown promising effects in weight management by PPAR-γ activation [146].

Adipogenesis, the formation of new fat cells, is regulated by transcription factors such as PPAR-γ, SREBP-1, and C/EBP-α. Peptides derived from macroalgal species, including *Plocamium telfairiae* (Rhodophyta) and *Undaria pinnatifida* (Phaeophyceae), downregulate these adipogenic regulators, thereby limiting the formation of new adipocytes and reducing further fat accumulation [146].

Thermogenesis is the process by which the body generates heat, often by burning fat. A critical player in this process is a mitochondrial protein called Uncoupling Protein 1 (UCP-1) [147]. UCP-1 is typically found in brown and beige fat cells, and it enables the cells to convert stored energy into heat rather than fat. Peptides (Ile-Ala-Glu, Phe-Ala-Leu, Ala-Glu-Leu, Ile-Ala-Pro-Gly, and Val-Ala-Phe) from *Spirulina/Limnospira* specie promote the upregulation of UCP-1, thereby enhancing the body’s thermogenic capacity [146]. Essentially, this means more calories are burned throughout the day, even at rest. The activation of UCP-1 supports overall energy expenditure, which may support weight management when combined with dietary and lifestyle interventions [148,149].

Fat accumulation doesn’t just come from dietary fats; it can also result from the body’s own fat synthesis pathways. One key enzyme involved in synthesizing fatty acids is Acetyl-CoA Carboxylase (ACC) [150]. This enzyme initiates the conversion of carbohydrates and other substrates into fatty acids, which are then stored as fat [146]. Peptides derived from *L. platensis* inhibit ACC activity, thereby reducing fatty acid synthesis and limiting lipid accumulation in hepatic and adipose tissues [151–153].

One of the most important regulators of energy balance in the body is an enzyme known as AMP-activated protein kinase (AMPK) [154]. When activated, AMPK works as an energy sensor, switching the body into a mode that conserves energy and promotes fat usage rather than storage [155]. Algal peptides help activate AMPK, leading to a cascade of beneficial metabolic effects. These include promoting the breakdown of fats (lipolysis), enhancing insulin sensitivity (which improves glucose uptake by cells), and increasing the translocation of GLUT-4, a glucose transporter that helps cells absorb glucose from the bloodstream. Altogether, these actions support a healthier metabolism and assist in gradual, sustainable weight management [146]. Several algal peptides have been identified to exert anti-obesity effects through the activation of AMP-activated protein kinase (AMPK). Notably, peptides derived from *Limnospira platensis*, *Auxenochlorella pyrenoidosa*, and the macroalgae *Ulva lactuca* have demonstrated such activity [115,119].

Adiponectin is a hormone secreted by fat cells that plays an essential role in regulating glucose and lipid metabolism. Algal peptides such as *BHD1028*, *BHD43*, and *BHD44* positively influence adiponectin signaling pathways through two specific receptors, AdipoR1 and AdipoR2 [156]. AdipoR1 activation stimulates AMPK, enhancing glucose uptake and fatty acid oxidation, thereby improving insulin sensitivity and energy expenditure. AdipoR2 activation promotes PPARα signaling, leading to increased fatty acid oxidation and reduced lipid accumulation in the liver and muscle tissues [157].

Our bodies rely on an enzyme called pancreatic lipase to break down dietary fats in the digestive system so they can be absorbed. When this enzyme is less active, less fat is digested and absorbed, and more is excreted from the body [158]. Algal peptides naturally inhibit pancreatic lipase, thereby decreasing the amount of dietary fat that gets absorbed [115]. This simple yet effective mechanism can help reduce overall calorie intake and fat storage, supporting weight loss without the need for severe dietary restrictions. The peptides, i.e. NPVWKRK and CANPHELPNK, derived from significantly reduced triglyceride accumulation due to pancreatic lipase inhibition [118]. This inhibition of fat absorption also leads to lower circulating triglyceride levels, which plays a crucial role in reducing the risk of atherosclerosis and other cardiovascular diseases. Peptides from *Auxenochlorella pyrenoidosa* demonstrated the ability to inhibit intestinal lipid absorption that helps to regulate lipid metabolism by lowering LDL cholesterol and triglyceride levels while increasing HDL cholesterol. Together, these properties highlight the dual role of algal peptides in promoting both weight management and cardiovascular health [115].

Another valuable mechanism involves the regulation of appetite and satiety. Some algal peptides stimulate the release of GLP-1 (Glucagon-Like Peptide-1), a gut hormone that helps control blood sugar levels and promotes a feeling of fullness after meals [97]. When GLP-1 levels are elevated, the stomach empties more slowly, and hunger signals are suppressed, which naturally reduces food intake [141]. Algal peptides derived from *Palmaria palmata* can make it easier for individuals to maintain a calorie deficit by promoting satiety [97].

Notably, findings from human intervention studies reinforce the translational significance of these weight-modulating effects. Clinical trials involving *Spirulina/Limnospira* supplementation have reported modest but consistent improvements in appetite regulation, reductions in triglyceride levels, and small decreases in body weight and BMI over intervention periods ranging from 8 to 12 weeks, particularly at doses around 2 g/day [159–161]. These outcomes are frequently accompanied by improved lipid profiles and reduced hunger sensations, aligning with the AMPK, pancreatic lipase inhibition, and GLP-1-mediated satiety mechanisms observed in preclinical studies. Similarly, *Chlorella* supplementation has been associated with improvements in waist circumference, hepatic fat markers, serum triglycerides, and metabolic enzyme profiles in individuals with metabolic dysfunction or fatty liver features [162,163]. Although the magnitude of weight loss in human studies is generally smaller than that observed in controlled animal models, these findings support a contributory role for algal-derived bioactives in weight management when used as part of broader dietary and lifestyle interventions.

Continued research into their bioavailability, long-term efficacy, and synergistic effects with diet and lifestyle interventions will be critical for their future application in managing cardiovascular diseases and obesity through clinical and functional food settings. Table 5 offers a detailed summary of significant peptides derived from algae, their respective sources, and the biological mechanisms through which they may aid in obesity prevention and treatment.

**Table 5.** Anti-obesity effects of algal peptides.

Peptides	Species name	Mechanism	Model (*in vitro*/Animal/Human)	Dose/ Concentration	Observed Effect	References
NALKCCHSCPA, LNNPSVCDCDCMMKAAR, NPVWKRK, and CANPHELPNK	*Limnospira platensis* (Cyanophyceae)	Pancreatic lipase inhibition, PPAR-γ Activation	*in vitro* (3T3-L1 preadipocytes)	3–5 kDa fraction: pepsin digest; purified peptides tested at 600 μg/mL	Lipase inhibition 72%; preadipocyte proliferation inhibited 32–60%; triglyceride accumulation decreased 19.5–23.7% (NPVWKRK & CANPHELPNK)	[118]
PP1 (Leu-Leu-Val-Val-Tyr-Pro-Trp-Thr-Gln-Arg)	*Auxenochlorella pyrenoidosa* (Chlorophyta)	Pancreatic lipase inhibition	*in vitro* (3T3-L1 preadipocytes)	Lipase Assay: 200 μg/mL; 3T3-L1 cells: 600 μg/mL	Decreased intracellular triacylglycerol by 27.9%; inhibited lipid accumulation and fatty acid synthesis *via* C/EBPα, SREBP-1c, PPARγ, AMPKα	[115]
PPPH peptides	*Palmaria palmata* (Rhodophyta)	GLP-1 stimulation	*in vitro* (BRIN-BD11, GLUTag, STC-1 cells); Animal (streptozotocin-induced diabetic mice)	0.02–2.5 mg/mL (*in vitro*); acute administration in overnight-fasted mice	Increased insulin secretion, enhanced GLP-1 and GIP release, improved glucose tolerance, promoted satiety, DPP-4 inhibition retained after simulated gastrointestinal digestion	[97]
Peptides derived from the brown seaweed *Undaria pinnatifida*	*Undaria pinnatifida* (Phaeophyceae)	PPAR-γ Activation, UCP-1 Expression, ACC Inhibition, AMPK Activation	Not specified (review-based)	Not specified	Reduced white adipose tissue, improved lipid metabolism, increased thermogenesis	[146]
SPP &SPPH	*Spirulina/Limnospira* sp. (Cyanophyceae)	Modulation of Lipid Metabolism Genes and Metabolic pathways (PPAR signaling, adipocytokine signaling, AMK)	High-fat diet-fed mice (C57BL/6J)	2 g/kg/day, oral gavage	Reduced body weight by 39.8 ± 9.7%; lowered serum glucose by 23.8 ± 1.6%; decreased total cholesterol by 20.8 ± 1.4%	[117]
LLVVYPWTQR	*Auxenochlorella pyrenoidosa* (Chlorophyta)	AMPK Activation	3T3-L1 adipocytes (*in vitro*)	600 μg/mL	Reduced intracellular triacylglycerol accumulation by 27.9%; inhibited fatty acid synthesis	[115]
Ile-Ala-Glu, Phe-Ala-Leu, Ala-Glu-Leu, Ile-Ala-Pro-Gly, and Val-Ala-Phe	*Spirulina platensis* (Cyanophhyceae)	PPAR-γ Activation, UCP-1 Expression, ACC Inhibition, AMPK Activation	Not specified (review-based)	Not specified	potential anti-obesity effects *via* modulation of lipid metabolism and thermogenesis	[3]
RuBisCO protein	*Ulva lactuca* (Chlorophyta)	ACC Inhibition, AMPK Activation	in silico	N/A	Identification of numerous putative bioactive peptides with anti-obesity, antihypertensive, antioxidant, and metabolic regulatory potential	[119]
Val-Trp (VW), Val-Tyr [61], Lys-Tyr (KY), Lys-Trp (KW), Ile-Tyr (IY), Ala-Pro (AP), Val-Ile-Tyr [24], Leu-Lys-Pro (LKP), Gly-Pro-Leu (GPL), Ala-Lys-Lys and Val-Ala-Pro (VAP)	*Undaria pinnatifida* (Phaeophyceae)	Suppression of Adipogenesis by down regulation of both PPARγ and C/EBPα expression	Human white pre-adipocytes (HWP)	Not specified	AP, VAP, AKK affected proliferation; KW, VW reduced differentiation viability; GPL, IY decreased final lipid content, GPDH activity, and mRNA of adipocyte markers (aP2, GLUT4, LPL, AGT)	[164]
LSSATSAPS and AGLYGHPQTQEE	*Chlorella sorokiniana* (Chlorophyta)	AMPK Activation, Glucose uptake stimulation, Antioxidant properties	*in vitro* (enzyme assays and molecular docking)	Hydrolysate <3 kDa, fraction C80%	Highest DPPH radical scavenging (22.04% inhibition); peptides predicted to bind ROS1 receptor	[165]

## 4. Commercialized algal protein-based nutraceuticals

Algal proteins and peptides are commonly marketed as powders, capsules, or tablets and are valued for their broad spectrum of health benefits, including supporting cardiovascular health, boosting the immune system, reducing inflammation, and providing potent antioxidant protection [6]. *Chlorella* and *Spirulina/Limnospira* are especially known for their high protein content and are frequently utilized as dietary supplements. Additionally, other algae such as *Haematococcus lacustris* (formerly *Haematococcus puvialis*) (Chlorophyta) and *Limnospira platensis* are celebrated for their rich content of compounds like astaxanthin, which offer specific therapeutic benefits. Table 6 presents a comprehensive overview of commercially available nutraceuticals derived from various algal species, emphasizing the role of algal proteins [169,175–178].

**Table 6.** List of commercialized nutraceuticals available in different parts of the world.

+	Algal protein	Uses	Company	Origin	References
*Spirulina/Limnospira* (Cyanophyceae)	*Spirulina/Limnospira* protein, Phycocyanin	Nutritional supplement	Earthrise Nutritionals, LLC	California, USA	[166,167]
*Chlorella* (Chlorophyta)	*Chlorella* Protein Concentrate	Nutritional supplement	Earthrise	USA	[166,167]
*Haematococcus lacustris* (Chlorophyta), *Spirulina/Limnospira* (Cyanophyceae)	*Spirulina/Limnospira* protein, Phycocyanin	Antioxidant, anti-inflammatory	Cyanotech Corporation	Hawaii, USA	[166,167]
*Limnospira platensis* (Cyanophyceae), *Haematococcus pluvialis* (Chlorophyta)	*Spirulina/Limnospira* protein, Phycocyanin	Antioxidant	BlueBioTech Int. GmbH	Germany	[168]
*Chlorella* (Chlorophyta), *Spirulina/Limnospira* (Cyanophyceae)	*Spirulina/Limnospira* protein, *Chlorella* protein	General health, immune boost	E.I.D., Parry (India)c Limited	India	[169]
*Spirulina/Limnospira* (Cyanophyceae), *Neopyropia yezoensis* (Rhodophyta), *Chlorella vulgaris* (Chlorophyta)	*Spirulina/Limnospira* protein, *Chlorella* protein	Cardiovascular diseases, hypertension	Cyanotech	USA	[170]
*Wollea saccata* (Cyanophyceae), *Leptolyngbya fragilis* (Cyanophyceae), *Limnospira plantensis* (Cyanophyceae)	*Spirulina* /*Limnospira* protein	Plant fungal diseases	Greenwave	USA	[171]
*Gracilaria* sp. (Rhodophyta), *Microcystis aeruginosa* (Cyanophyceae)	*Gracilaria* protein, *Microcystis* protein	Skin cancer	Fucoidan Force	Japan	[172]
*Chlorella vulgaris* (Chlorophyta)	*Chlorella* protein	Immune system support, antioxidant properties	Sun *Chlorella* Corp., E.I.D Parry	Japan, India	[173]
*Nannochloropsis* (Eustigmatophyceae)	*Nannochloropsis* protein	Nutritional supplements, aquaculture feed	Veramaris	France	[174]

## 5. Life-cycle and economic outlook

Building on the scientific evidence and emerging commercial applications of algal peptides, it is essential to consider the broader environmental, economic, and operational context for their production. The production of algal peptides and protein ingredients holds promise not only for health and nutrition but also for sustainable food systems, provided key environmental and economic challenges are addressed. Microalgae require minimal arable land and water resources compared to terrestrial crops, making them attractive alternatives to soybean or animal proteins; microalgal protein yields of 4–15 t per hectare per year far exceed those of soybeans and legumes, highlighting efficiency gains in land use and protein productivity [179].

Recent Life Cycle Assessment (LCA) studies emphasize that these sustainability advantages depend strongly on cultivation design, energy inputs, and downstream processing intensity. Systems powered by renewable energy and coupled with wastewater nutrient recovery demonstrate lower greenhouse gas emissions, whereas energy-intensive drying, cooling, and harvesting substantially increase the environmental burden. Photobioreactors provide culture stability but generally have higher energy demand, while open-pond systems are cheaper but face productivity variability and contamination risk [180].

Their capacity to sequester CO_2_ through photosynthesis and utilize industrial flue gases or wastewater nutrients offers potential climate benefits by reducing net greenhouse gas emissions and supporting nutrient recycling. Life-cycle assessments similarly point to lower carbon footprints for microalgal protein production compared with conventional animal proteins when renewable energy and CO_2_ recycling are integrated [181]. However, true carbon efficiency is most evident when algal cultivation is embedded in circular-economy systems, such as industrial symbiosis or wastewater treatment co-location [182].

Despite these advantages, economic barriers remain significant. Microalgal biomass can be produced at costs ranging from approximately $1.8–8.1 per kg dry weight when integrated with wastewater treatment systems, although costs increase when processing for protein and peptide isolates [183]. These production costs exceed those of conventional plant proteins, which typically cost a few dollars per kilogram, reflecting higher energy, harvesting, and downstream processing demands [181]. Techno-Economic Analysis (TEA) further shows that downstream processing such as harvesting, cell disruption, hydrolysis, and purification contributes the largest fraction of total production cost, highlighting it as the key economic bottleneck. Production becomes more feasible when peptides are generated as part of a multi-product biorefinery that also recovers pigments, oils, or other high-value components [184,185].

Algal systems can enhance sustainability and reduce costs by leveraging open pond cultivation, wastewater nutrient recycling, and co-product generation such as pigments and lipids, although pond systems trade off lower capital costs for increased contamination risk and variable yields [181]. Downstream processing, such as harvesting, breaking the cells, extracting, and purifying peptides, is a major cost and bottleneck, so more efficient and low-energy methods are needed [183]. Economies of scale also play a major role, with larger biorefinery facilities able to distribute capital and operating costs across multiple product streams [186].

From a supply-chain perspective, biomass production increasingly occurs in emerging markets such as Southeast Asia and East Africa, where seaweed and microalgae farming support local livelihoods. However, much of the value addition currently happens in high-income countries. Developing regional processing capacity, alongside frugal innovations suitable for low-resource settings, may enable more equitable value capture while supporting nutrition security [187].

Algal peptide production also aligns with multiple Sustainable Development Goals (SDGs). High-protein microalgal biomass can contribute to SDG 2 (Zero Hunger) by supplementing local protein supplies, while nutrient recycling during cultivation helps achieve SDG 6 (Clean Water) by reducing freshwater pollution. Co-products such as biofuels and residual biomass support SDG 7 (Affordable and Clean Energy), and the inherent CO_2_ fixation of microalgae contributes to SDG 13 (Climate Action) by lowering net greenhouse gas emissions [188,189]. Ethical sourcing frameworks such as the Nagoya Protocol further emphasize equitable benefit-sharing from marine genetic resources, supporting SDGs related to poverty reduction, gender empowerment, and responsible ocean stewardship [190].

This combination of environmental benefits and economic challenges frames the broader outlook for algal peptide production and underscores the importance of large-scale, integrated systems to improve feasibility. While current costs remain higher than those for traditional protein sources, advances in cultivation technologies, energy efficiency, and economies of scale are expected to reduce these gaps, supporting the positioning of algal peptides as a sustainable and viable option within future protein and nutraceutical markets [181].

**Figure 4.**
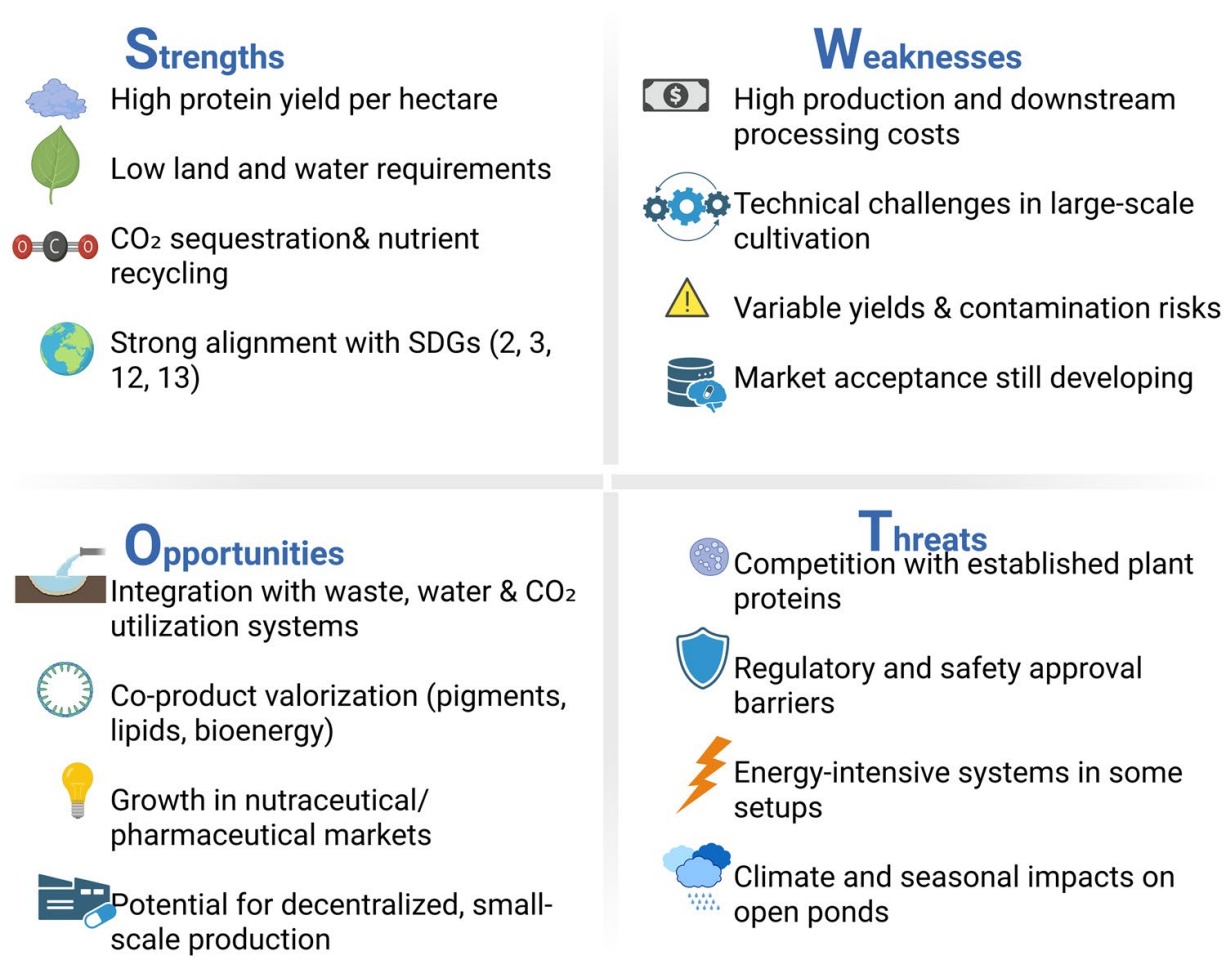
SWOT analysis of algal peptides.

Figure 4 demonstrates the summary of the strengths, weaknesses, opportunities, and threats associated with large-scale production of algal peptides and protein ingredients, highlighting sustainability benefits, technical constraints, and commercialization potential.

## 6. Critical insights and challenges

While the environmental and economic potential of algal peptide production is promising, several scientific and technological challenges still hinder its full translation to industrial scale. In recent years, the health-promoting benefits of algal peptides have gained considerable attention, with increasing evidence supporting their antihypertensive, antidiabetic, antioxidant, and anti-inflammatory effects. However, despite this growing interest, the available data remain largely confined to laboratory and animal models. Translating these promising findings into commercially viable applications presents several ongoing challenges.

Although it is estimated that between 200,000 and 800,000 microalgal species exist globally, only around 30,000 have been identified and studied to some extent, with just a small fraction evaluated for their health-related potential [3,111]. To date, research has focused primarily on a handful of well-known genera, including *Spirulina/Limnospira*, *Chlorella*, *Haematococcus*, *Dunaliella*, and *Nannochloropsis* [191]. This limited taxonomic coverage highlights the need for broader exploration and utilization of under-researched algal species in the context of health and nutrition.

Furthermore, research on algal bioactive peptides has been disproportionately centered on microalgae, while macroalgae (seaweeds) remain comparatively underexplored despite their long history of dietary use and favorable production characteristics [73,192]. This imbalance is partly attributable to the generally lower protein content of macroalgae, the structural complexity of their polysaccharide-rich cell walls that limits protein extraction efficiency, and pronounced species- and season-dependent variability, which together complicate peptide isolation and standardization [193–195]. Macroalgae have traditionally been consumed in many coastal and Asian populations and can be cultivated at relatively low cost using open-water farming systems that do not require arable land, freshwater, or energy-intensive infrastructure [176,192]. In contrast, microalgae cultivation often relies on controlled photobioreactors or pond systems with higher capital and operational costs [176]. A more balanced evaluation of macro- and microalgal systems is therefore necessary to identify context-appropriate solutions for different production environments, particularly in low- and middle-income regions where affordable, scalable, and locally adaptable protein and peptide sources could contribute meaningfully to nutrition security.

The isolation of purified peptides from algal biomass presents significant technological obstacles. One of the major challenges in this regard is the complexity of protein extraction from algae [176]. Unlike plant-based proteins, algal proteins are intracellular and protected by rigid cell walls composed of resistant polysaccharides, which makes them more difficult to access [196]. Releasing these proteins requires physical or chemical disruption techniques, and even then, only a limited portion of the total protein content is typically recovered [3]. Once extracted, the production of bioactive peptides involves several downstream steps, including enzymatic hydrolysis, centrifugation, ultrafiltration, and purification. These processes are resource-intensive and demand considerable time, specialized equipment, and energy input [197]. While innovative methods such as bead milling, pulsed electric fields, and ultrasonication have shown some success in improving extraction efficiency, they remain economically challenging to implement at an industrial scale [198–200]. Therefore, optimizing stable protein extraction methods remains essential to fully harness the bioactive potential of algal peptides, especially as interest grows in clean-label and sustainable functional food applications [201].

Another critical limitation lies in the absence of standardized protocols for protein hydrolysis and peptide characterization. Differences in enzyme selection, hydrolysis duration, and analytical platforms often lead to inconsistent activity results, making cross-study comparison and reproducibility difficult. Establishing harmonized experimental workflows will be crucial to advance this field beyond proof-of-concept studies.

Another key issue is their uncertain bioavailability. Although many peptides have demonstrated promising biological activity *in vitro*, their behavior within the human digestive system is not fully understood [202]. During digestion, peptides may break down or be absorbed in altered forms, thereby reducing their bioactivity and therapeutic potential [203,204]. Strategies such as peptide modification, encapsulation, and advanced delivery systems are being explored to enhance stability and absorption; however, further investigation is necessary to validate their effectiveness [205]. Moreover, the field still lacks a substantial body of clinical data. Most of the current evidence is derived from *in vitro* assays or animal models, which, although informative, do not necessarily translate to human outcomes [202]. Until robust clinical trials are conducted, regulatory approval and consumer trust in peptide-based formulations will remain limited.

In addition, allergenicity assessment remains an underexplored challenge in algal peptide research. While food allergies directly attributed to algae are rare, systematic allergenicity testing of purified peptides and standardized risk evaluation protocols are still lacking, particularly for novel or underutilized algal species.

Along with scientific and technical challenges, the question of consumer acceptance must also be addressed. Protein hydrolysates and algal peptides often have strong flavors, marine-like odors, or unfamiliar colors, which can negatively affect consumer perception, especially in regions where algae are not traditionally consumed [201,206]. Addressing these sensory concerns requires thoughtful formulation strategies, such as the use of flavor-neutral blends, masking agents, or incorporation into familiar food formats [207,208]. At the same time, building consumer awareness around the safety, efficacy, and environmental benefits of algal peptides is equally important. Transparent communication and education efforts will be essential to fostering consumer confidence and supporting market adoption.

Addressing these biological, technical, and perceptual barriers requires clearly defined R&D priorities, including the development of standardized extraction and hydrolysis protocols, systematic allergenicity testing, improved strategies for gastrointestinal stability, and well-designed human clinical trials, areas explored in the following section.

## 7. Technological innovations and emerging applications of algal peptides

To address the limitations identified earlier, recent technological advancements are transforming the discovery, characterization, and application of algal bioactive peptides by accelerating bioactivity prediction, targeted hydrolysis, stability assessment, and therapeutic profiling. There is enhanced prediction of peptides, targeted hydrolysis, stability, and therapeutic efficacy being achieved with the help of modern computational tools, formulation technologies, and synergistic bioactive profiling.

### 7.1. Bioactivity screening and prediction of peptide AI-driven tools

One of the major constraints in algal peptide research is the limited exploration of algal biodiversity and the high cost of experimental peptide screening. Biological informatics platforms based on machine learning and artificial intelligence (AI) have transformed the field of peptide studies by allowing predictions of bioactivity, toxicity, physicochemical properties, and possible health impact within hours. Libraries of bioactive peptide sequences obtained *via* proteolysis of food proteins are widely available in databases like BIOPEP-UWM and contain modules of in silico proteolysis, activity prediction, and structure-function analysis [209]. The ML-based tool for ranking peptides, named PeptideRanker, can also assist with high-throughput screening, ranking peptides according to their likelihood to be biologyically active so that researchers prioritize sequences to be synthesized chemically or tested *in vivo* [210].

The AlphaFold and other structure prediction technologies have also transformed the study of peptides by providing precise three-dimensional representations of peptide conformations and the interactions of peptides with their target proteins. It is also useful in the study of the algal peptide-biomarker interactions, such as that between algal peptides and ACE (angiotensin-converting enzyme) or DPP-IV (dipeptidyl peptidase-IV) [211].

Software programs like AutoDock, GOLD, and Glide use docking simulations to predict the binding of algal peptides to target protein receptors. They provide a three-dimensional model of peptide-ligand interactions, scoring the binding affinity and identifying the most thermodynamically favorable conformation [212,213].

These AI applications save lab expenses and time by a significant margin, simulate of enzymatic digestion patterns, the peptide cleavage sites by proteins like pepsin, trypsin, and alcalase, and discover of antioxidant, ACE-inhibitory, antidiabetic, or immunomodulatory fragments prior to experimental confirmation. Such in silico-directed approaches have gained increasing popularity in recent studies of algal proteins (e.g. *Limnospira platensis*, *Chlorella vulgaris*, and *Ulva lactuca*) to guide the discovery process faster and maximize the specificity of peptide selection [119,214,215].

### 7.2. Nanoencapsulation to achieve stability, targeted therapy, and bioavailability

Although algal peptides may have high potential bioactivity, they are usually limited by their potential to be destroyed by the gastrointestinal tract, their instability during processing, and their inability to be absorbed into the systemic environment. Nanoencapsulation technologies such as nanoemulsions, liposomes, biopolymer nanoparticles, and nanofibers are there as effective options to protect against the degradation of peptides in addition to offering controlled release. Liposome-based encapsulation is also proven to enhance the oral stability of antioxidant peptides and enhance intestinal permeability [185]. Nanocarriers made out of biopolymers (e.g. chitosan-alginate nanoparticles) increase the solubility of peptide molecules and enable pH-dependent release in the intestine, which is of particular importance in the delivery of algal peptides with antihypertensive or antidiabetic properties [216]. In the case of algal-derived peptides in particular, nanoencapsulation has allowed enhancement of antioxidant effect and increased circulation, which can be used in the context of functional foods and nutraceutical [217].

### 7.3. Synergy effects with other algal bioactives

Microalgae are not only a good source of proteins but also of complementary bioactives like carotenoids (β-carotene, astaxanthin), polyphenols, phycobiliproteins, sulfated polysaccharides, and omega-3 fatty acids. The developing studies indicate that the synergistic effects of peptides and these compounds are effective in McLaughlin et al. [97] imp roving antioxidant activity, anti-inflammatory activity, metabolic homeostasis, and immune-enhancing effects [141,185]. Indicatively, antioxidant peptides of *Spirulina/Limnospira* have been demonstrated to synergize with phycocyanin, enhancing the scavenging of reactive oxygen species and regulating inflammatory responses [185]. This synergy underscores the possibility of creating multi-component formulations based on algae instead of peptide-based formulations.

Collectively, these innovations bridge laboratory research with industrial translation, enabling the scalable use of algal peptides in health-promoting applications.

## 8. Regulatory and safety considerations

### 8.1. Heavy metal risk in algae

Although algae are widely recognized for their nutritional value, providing essential trace elements, minerals, vitamins, antioxidants, and bioactive compounds, their ability to bioaccumulate contaminants from the surrounding environment makes them susceptible to heavy metal uptake, posing potential risks to human health [218]. Seaweeds and microalgal species growing in polluted waters, especially those near industrial zones, mining areas, poorly managed sewage systems, or regions exposed to natural geological activity, can accumulate significant concentrations of toxic metals such as arsenic, cadmium, lead, mercury, and aluminum [219,220]. These contaminants derive primarily from anthropogenic sources, including mining effluents, industrial discharges, and urban pollution, and quickly integrate into algae tissues before entering the broader food chain [221,222]. Variability in heavy metal concentrations is influenced not only by environmental contamination but also by species-specific uptake mechanisms [223]. Environmental factors such as salinity, pH, sunlight exposure, nutrient availability, water currents, and seasonal changes further contribute to large geographic disparities in contamination levels [224,225]. For example, arsenic concentrations in the brown seaweed *Undaria pinnatifida* collected from different coastal regions of Eastern China ranged from 19.4 to 34.2 mg/kg dry weight, while *Neopyropia yezoensis* samples showed As levels of 24.5–32.0 mg/kg [226]. On a broader scale, *Laminaria ochroleuca* exhibited average As concentrations of 76.8 mg/kg in China, 52.5 mg/kg in Korea, and 41.1 mg/kg in Spain [227], demonstrating how regional ecological conditions and species traits magnify disparities in heavy metal levels. These variations complicate risk assessment and highlight the need for precaution in consumption, particularly when algae are consumed frequently or in concentrated forms such as supplements [227].

The health implications of heavy metal exposure through algae consumption are significant. Toxic metals such as As, Cd, Pb, and Hg are associated with cancer, cognitive impairment, cardiovascular diseases, and other chronic health conditions [228,229]. In addition to heavy metal contamination, iodine accumulation remains a critical concern, particularly in seaweeds [230]. Although iodine is essential for thyroid hormone synthesis and beneficial in preventing deficiencies, excessive intake has been linked to thyroid dysfunction, autoimmune thyroiditis, and increased risk of thyroid cancers such as papillary carcinoma [231,232]. Epidemiological studies from Japan, where seaweed contributes approximately 80% of dietary iodine, show that frequent seaweed consumption (3–4 times per week or more) correlates with a higher risk of thyroid cancer, with hazard ratios increasing from 1.37 for moderate consumption to 1.52 for daily intake [233]. A long-term follow-up study reported a 1.71-fold increased risk of papillary carcinoma among the daily seaweed consumers [234]. Conversely, some studies, particularly those in Korea, have observed protective associations between high seaweed consumption and lower thyroid cancer prevalence [235]. These conflicting findings emphasize the need for species-specific investigations to determine safe iodine consumption thresholds, recognizing that iodine concentrations vary substantially across seaweed varieties.

Regulatory frameworks governing heavy metal contamination in algae remain inconsistent globally. In the European Union, Commission Regulation (EC) No. 1881/2006 sets general limits for heavy metals in foods but lacks specific maximum levels for cadmium or inorganic arsenic in microalgae. Regulation (EC) No. 396/2005 establishes a mercury limit of 0.01 mg/kg, while cadmium in food supplements is limited to 3.0 mg/kg. General maximum limits for mercury and lead are 0.1 mg/kg and 3.0 mg/kg, respectively. Although EU Regulation (EC) No. 1333/2008 lists multiple algal-derived food additives (E401–E407a) [236], only France has established explicit seaweed-specific limits for metals and iodine. Other countries, such as Germany, rely on broader food safety rules, thereby leaving regulatory gaps for algae consumed directly. In Asia, standards vary widely; China has set limits for inorganic arsenic and lead in selected seaweed products but lacks complete thresholds for Cd, Hg, and iodine. South Korea regulates Cd and Pb only in certain species, Hong Kong sets limits solely for inorganic As, and Taiwan imposes broader yet still variable heavy metal limits across seaweed categories. These disparities reflect the need for harmonized, species- and region-specific global standards.

Mitigation strategies increasingly emphasize improved coastal monitoring, avoidance of harvesting from contaminated waters, and the adoption of post-harvest treatments such as boiling and soaking, which can reduce certain metal concentrations [237]. Equally important is the requirement for transparent and accurate labeling of species, origin, processing methods, iodine content, and potential contaminant levels. Clear labeling, including recommended serving sizes, is essential for consumer safety, particularly in regions where algae form a dietary staple or where unregulated markets and informal sales channels increase the likelihood of exposure to unsafe products. Given the expanding global reliance on algae for food, supplements, and functional ingredients, comprehensive monitoring, robust regulation, and transparent communication are critical to ensuring safe consumption and preventing heavy metal-related health risks.

### 8.2. GRAS status and EU regulations

Global regulatory bodies and food-standards authorities oversee the permissible uses of algae in products intended for human consumption. In many regions, including the European Union, algal species and their derivatives are assessed individually rather than under a unified regulatory framework. Depending on the intended application, algae may be classified across multiple sectors, such as conventional foods, dietary supplements, food additives, animal feed products, nutraceuticals, cosmetics, packaging materials, fertilizers, biostimulants, and biofuels [238].

In the United States, the Food and Drug Administration (FDA) assigns Generally Recognized as Safe (GRAS) status to substances whose safety for human consumption has been established through a history of use or sound scientific evidence [2,239]. Any organism intended for inclusion in foods must either achieve GRAS status or receive FDA pre-market approval if classified as a new food additive [240]. Obtaining GRAS approval is often a lengthy and costly process, and only a limited number of microalgal species have received this designation to date. These include *Chlorella* sp., *Spirulina/Limnospira* sp., *Dunaliella* sp., *Schizochytrium* sp., *Haematococcus* sp., *Porphyridium purpureum* (formerly *Porphyridium cruentum*) (Rhodophyta), and *Crypthecodinium cohnii* (Dinophyceae). In addition, the FDA has granted GRAS status to oils derived from *Schizochytrium* and *Ulkenia* (fungi), as well as to certain whole-protein and lipid ingredients produced from *Chlorella* sp. [236].

Globally, similar pre-market safety frameworks exist. Canada regulates novel foods through Health Canada [241], while Australia and New Zealand follow the Food Standards in Australia and New Zealand (FSANZ) regulations requiring explicit authorization for non-traditional foods [242]. In China, “novel foods” are regulated as “new food raw materials,” with safety oversight by the National Health Commission; microalgae are mainly regulated as novel foods, whereas many seaweeds are considered traditional foods [243].

In the European Union, algae for human consumption fall under the General Food Law (EU 178/2002) [244] and the Novel Food Regulation (EU 2015/2283) [245]. Algal species without documented EU consumption before 15 May 1997 require pre-market authorization, while traditionally consumed non-EU species may follow a simplified notification route [246]. Ongoing regulatory gaps related to algae as raw materials led to the formation of CEN/TC 454 in 2017, tasked with developing standards for algal biomass, extracts, and purified compounds, including terminology, processing methods, and quality criteria [238].

National variations also exist. Some EU member states have implemented their own guidelines, and in certain cases, non-approved algal species are already being sold for food use. France, for instance, established a specific regulatory framework in 1990 to permit seaweed consumption beyond what is classified as novel food at the EU level [247,248]. Regarding edible species, more than 150 types of algae are known to be consumed in Europe, mainly seaweeds (86%), with a smaller proportion being microalgae and Cyanophyceae (14%). However, only about 30 species are formally authorized as novel foods [249]. Many edible seaweeds widely consumed globally, especially in Asia, are not listed in EU catalogues but are still imported to meet market demand.

### 8.3. Allergen labelling and safety testing for food applications

Food allergies directly attributable to algae are rare and remain poorly characterized in the literature. To date, only a single clinical case linked specifically to nori consumption has been formally reported [250]. Although microalgae such as *Chlorella* sp. and certain Cyanophyceae have been identified in household dust, some cyanobacterial species are known to produce irritant compounds associated with dermatitis and respiratory inflammation. These reactions arise from environmental exposure rather than from consumption of processed algal food products [251].

In practice, most allergenic risks associated with edible algae originate from external biological contaminants rather than from the algae themselves. Seaweeds are typically cultivated and harvested in open marine or estuarine environments that host crustaceans such as amphipods, shrimp, and crabs, which are well-established triggers of immunoglobulin E-mediated food allergies. For instance, studies evaluating the allergenicity of nori sheets identified amphipod-derived tropomyosin as the principal allergen; however, detected levels were minimal, suggesting that hypersensitivity reactions following ingestion would be uncommon [252]. Despite advances in cultivation and processing that reduce contamination risk, incidental inclusion of crustacean fragments may still occur, posing a concern for highly sensitized individuals and functioning as hidden allergens [253].

The European Food Safety Authority (EFSA) applies a structured, tiered approach to assess the allergenicity of novel foods derived from microalgae, in accordance with its latest guidance [254]. This framework includes a comprehensive literature review and evaluation of phylogenetic relationships, bioinformatic analyses to predict potential cross-reactivity, *in vitro* human serum specific IgE binding assays and, where necessary, human studies such as skin prick or patch tests. Allergenicity assessments typically consider protein content, history of safe use, prior EFSA evaluations, protein identification by LC-MS/MS, the presence of allergens subject to mandatory labeling, and *in vivo* testing. Although EFSA has generally concluded that allergenicity risks associated with microalgal novel foods are low [255], it has also highlighted specific cases where allergic reactions may occur, particularly in shrimp-allergic individuals due to cross-reactivity, emphasizing the importance of rigorous pre-market evaluation and appropriate allergen labeling [256].

Given these potential risks, transparent and precautionary allergen labeling is essential for the safe market translation of algal products. Accurate labeling not only alerts consumers, especially those with known shellfish allergies, to possible contamination but also ensures regulatory compliance and builds consumer trust. As an example, Japan mandates the inclusion of shrimp allergen warnings on nori products to indicate possible crustacean contamination. Such regulations underscore the importance of comprehensive allergen labeling in facilitating safe commercialization and broader acceptance of algae-derived foods.

## 9. Future perspectives

Building upon the technological innovations and challenges discussed above, future research on algal peptides should prioritize translational studies that validate their efficacy, safety, and scalability for real-world applications. To strengthen the bridge between laboratory findings and applied nutrition, upcoming studies should focus on long-term randomized clinical trials that establish dose–response relationships, absorption kinetics, and measurable physiological outcomes in diverse populations.

Despite ongoing challenges, the field of algal bioactive peptides is advancing rapidly, driven by innovations in bioinformatics, omics technologies, and machine learning. These tools enable precise prediction and modeling of peptide bioactivity based on amino acid sequences, facilitating the rational design of peptides tailored to specific therapeutic targets and enhancing both efficiency and functionality within development pipelines [257]. Integrating proteomics, metabolomics, and transcriptomics with AI-based peptide discovery could further unravel structure-function relationships and reveal new bioactive motifs with superior stability or selectivity. Alongside these technological strides, attention is increasingly turning toward underexplored algal species, particularly extremophiles, organisms that thrive in harsh environmental conditions. These unique species offer a largely untapped reservoir of peptides with potentially novel structures and bio-functionalities, expanding the scope for discovery beyond well-characterized taxa [257,258]. As the search for alternative protein sources intensifies, these underexplored algae may prove valuable in diversifying both the functional and nutritional landscape of future foods.

Simultaneously, the dual role of algal proteins as both bioactive and techno-functional agents, serving as emulsifiers, gelling agents, and stabilizers, broadens their utility in modern food systems [141,201]. This versatility makes them particularly suitable for integration into innovative product formats such as functional beverages, fortified snacks, or meal replacements. Notably, algae-derived bioactive peptides have demonstrated various health benefits, including anti-diabetic, anti-inflammatory, and antihypertensive activities. These properties suggest potential applications in personalized nutrition strategies aimed at managing conditions like diabetes and hypertension. Advancements in bioinformatics and silico methods, such as quantitative structure-activity relationships (QSAR) and molecular docking, facilitate the identification and design of specific peptides tailored to individual nutritional needs [257,259].

To ensure sustainable development, future work should emphasize industrial scalability through improved bioreactor efficiency, the use of low-cost substrates, and the integration of cultivation systems with wastewater treatment and carbon capture. Embedding algal peptide production into circular bioeconomy models will help lower production costs while minimizing environmental impact. In parallel, integrating microalgae cultivation with wastewater treatment and carbon dioxide sequestration aligns with circular bioeconomy principles, offering a sustainable model for producing high-value bioproducts, including bioactive peptides [260]. Equally important is the development of policies and consumer education programs that address labeling, allergenicity, and perceived safety concerns. Strong collaboration between scientists, regulatory bodies, and industry stakeholders will be essential to build public confidence and support market adoption.

However, as the field progresses toward the commercialization of peptides derived from novel or underutilized algal species, ensuring consumer safety remains paramount. Comprehensive toxicological evaluations and allergenicity assessments are critical, alongside the development of standardized protocols for peptide extraction, characterization, and regulatory compliance. These measures are essential to support the commercial scalability and global acceptance of algal-derived functional ingredients. Ultimately, by bridging mechanistic understanding, technological innovation, and regulatory readiness, algal peptides could emerge as a cornerstone of sustainable nutrition, contributing not only to human health but also to environmental resilience and the United Nations Sustainable Development Goals (SDGs).

## 10. Conclusion

In conclusion, the future of algal peptides lies not only in scientific discovery but also in the practical strategies adopted to bring them into mainstream food systems. Realizing their full potential will require continued innovation in extraction technology, formulation science, and clinical research, as well as regulatory harmonization. Addressing consumer expectations through thoughtful product design and transparent communication will be equally important. With sustained interdisciplinary collaboration, algal peptides could emerge as a vital component of a more health-conscious, sustainable, and protein-secure future.

## Data Availability

No datasets were generated or analyzed during the current study.
